# Non-linear mixed-effects modelling and population-based model selection for ^131^I kinetics in benign thyroid disease

**DOI:** 10.1186/s40658-025-00735-6

**Published:** 2025-04-08

**Authors:** Deni Hardiansyah, Ade Riana, Heribert Hänscheid, Ambros J. Beer, Michael Lassmann, Gerhard Glatting

**Affiliations:** 1 Medical Physics and Biophysics, Physics Department, Faculty of Mathematics and Natural Sciences, Universitas Indonesia, Depok, Indonesia; 2 Department of Nuclear Medicine, University Hospital Würzburg, Würzburg, Germany; 3 Department of Nuclear Medicine, Ulm University, Ulm, Germany; 4 Medical Radiation Physics, Department of Nuclear Medicine, Ulm University, Albert-Einstein-Allee 23, 89081 Ulm, Germany

**Keywords:** Akaike weight, Model selection, NLME, ^131^I, Benign thyroid disease, Radioiodine therapy

## Abstract

**Purpose:**

This study aimed to determine a mathematical model for accurately calculating time-integrated activities (TIAs) of target tissue in ^131^I therapy for benign thyroid disease using the population-based model selection and non-linear mixed-effects (PBMS-NLME) method.

**Methods:**

Biokinetic data of ^131^I in target tissue were collected from seventy-three patients at 2, 6, 24, 48, and 96 (*N* = 53) or 120 (*N* = 20) h after oral capsule administration with 1 MBq ^131^I. Based on the Akaike weight, the best sum-of-exponential function (SOEF) describing the biokinetic data was selected using PBMS-NLME modelling. Nine SOEF with three to six parameters (including the function from the European Association of Nuclear Medicine Standard Operational Procedure (EANM SOP)) were used. The fittings were repeated 1000 times with different starting values of the SOE parameters to find the optimal fit. Akaike weight was used to identify the performance of the best model from PBMS-NLME and the EANM SOP SOE function with individual fitting.

**Results:**

Based on the PBMS-NLME analysis, the SOEF $\frac{{\lambda_{1} }}{{\lambda_{2} + \lambda_{1} - \lambda_{3} }}\left( {e^{{ - \left( {\lambda_{3} + \lambda_{phys} } \right)t}} - e^{{ - \left( {\lambda_{1} + \lambda_{2} + \lambda_{phys} } \right)t}} } \right) + a_{1} e^{{ - \left( {\lambda_{1} + \lambda_{2} + \lambda_{phys} } \right)t}}$ was selected as the function most supported by the data. The Akaike weight of the best function was approximately 100%. The best SOEF from the PBMS-NLME approach shows a better performance in describing the biokinetic data of ^131^I in the thyroid gland than the function from the EANM SOP with individual fitting, based on the Akaike weight.

**Conclusions:**

The best mathematical model from the PBMS-NLME approach has one more free parameter than the EANM SOP function, which could lead to more accurate TIAs.

**Supplementary Information:**

The online version contains supplementary material available at 10.1186/s40658-025-00735-6.

## Introduction

In 2013, the European Association of Nuclear Medicine (EANM) Dosimetry Committee published a standardised operational procedure (SOP) for pre-therapeutic dosimetry before administering radioiodine therapy for benign thyroid disease [1]. This SOP outlined the method for calculating the appropriate therapeutic activity for radioiodine therapy in patients with hyperthyroidism or Graves’ disease. One pivotal step in determining the administered activity involves computing the individual time-integrated activity (TIA), which is performed by fitting a sum of exponentials function (SOEF) to the ^131^I retention data of the thyroid and subsequent calculation of the area under the curves [2, 3]. The EANM SOP employed a bi-exponential function comprising uptake and elimination phases based on a first-order pharmacokinetic compartmental model of the thyroid.

Although the bi-exponential function has seen frequent use in radioiodine therapy [1, 4, 5], there may be different choices for describing biokinetic data across various patient populations. An unresolved and critical matter in molecular radiotherapy (MRT) dosimetry pertains to selecting the fitting function; different modellers might use different functions for the same dataset, leading to reproducibility issues [6, 7]. Opting for an appropriate model is essential due to the substantial impact of the chosen function on the accuracy of calculated TIAs during MRT [6, 8, 9]. Recent research indicates that a population-based model selection and a non-linear mixed-effects (PBMS-NLME) model yields higher accuracy than an individual-based model selection for determining TIAs [10].

The PBMS-NLME approach is more reproducible as the model selection encompasses numerous suitable SOEF. This method applies to scenarios where pharmacokinetic information from heterogeneous data can be derived from a population and integrated for individual fits. Therefore, the objective of our study was twofold: first, to identify the optimal SOEF to improve TIA calculations in the context of ^131^I treatment for benign diseases, and second, to compare the performance of the SOEF from the EANM SOP using an individual fitting with the best SOEF from PBMS-NLME using population fitting, which serves as the reference standard.

## Materials and methods

### Biokinetic data

Data on target tissue retention (fraction of the administered activity in the thyroid gland a(t) as a function of time t) were collected from 73 patients (Age (66 ± 11) years, 19 males and 54 females) with Graves’ disease (12), toxic nodular goitre (59), and non-toxic goitre (2) at *t* = 2, 6, 24, 48, and 96 (*n* = 53) or 120 (*n* = 20) hours post oral administration of 1 MBq ^131^I [4]. Since one patient had no 6 h measurement, *N* = 364 retention data were included in the analysis. The measurement process involved a collimated 2 × 2″ NaI crystal equipped with a single-channel analyser. This setup underwent regular quality inspections, encompassing daily checks for background levels and sensitivity and weekly assessments of the energy spectrum. The biokinetic data were determined from count rates measured according to the EANM SOP [1] [4] at a distance of 35 cm above the neck and thigh. Assuming that in both measurements the count rates from the activity in the blood pool are identical, the difference in count rates represents the kinetics of the activity stored in the thyroid gland. This was done to rectify blood pool activity. The derived difference was standardised against the count rate obtained from the ^131^I capsule within a thyroid phantom before administration.

### Set of sums of exponential functions

First, we opted for a strategic approach by including a comprehensive list of sums of exponential functions (SOEFs). Rather than defining the compartmental model at the outset and deriving its analytical solutions, this approach allowed us to explore how many exponential terms were necessary to accurately determine the structure of the compartment model. Several constraints have been used for defining the SOEFs in this study, i.e. (1) only sums of exponentials functions (SOEFs) were considered [11], (2) all functions had to fulfil *f*(*t* = 0) = 0*,* and 3) radioiodine physical decays were considered as an exponential factor [6]. Therefore, SOEFs (two, three, and four terms) with three to six adjustable parameters with systematically increasing complexity [10] were used to describe the physical and biological processes of the ^131^I pharmacokinetics (Eq. (1–9)). The EANM-SOP (Eq. (2)) and an extended EANM-SOP (Eq. (5)) were included in the list. The latter, with an extra term for a contribution of the blood pool (Supplement, Equation (S3)), takes into account any residual count rate from the tissue activity.

1 $$a_{3a} \left( t \right) = a_{1} e^{{ - \left( {\lambda_{1} + \lambda_{phys} } \right)t}} - a_{1} e^{{ - \left( {\lambda_{2} + \lambda_{phys} } \right)t}}$$

2 $${a_{3b} \left( t \right) = \frac{{\lambda_{1} }}{{\lambda_{2} + \lambda_{1} - \lambda_{3} }}(e^{{ - \left( {\lambda_{3} + \lambda_{phys} } \right)t}} - e^{{ - \left( {\lambda_{1} + \lambda_{2} + \lambda_{phys} } \right)t}}) } \ \ \ \ \ \ \ \ \ {\text{EANM SOP}}$$

3 $$a_{4a} \left( t \right) = a_{1} e^{{ - \left( {\lambda_{1} + \lambda_{phys} } \right)t}} + a_{2} e^{{ - \left( {\lambda_{phys} } \right)t}} - \left( {a_{1} + a_{2} } \right) e^{{ - \left( {\lambda_{2} + \lambda_{phys} } \right)t}}$$

4 $$a_{4b} \left( t \right) = \left( {a_{1} + a_{2} } \right) e^{{ - \left( {\lambda_{phys} } \right)t}} - a_{1} e^{{ - \left( {\lambda_{1} + \lambda_{phys} } \right)t}} - a_{2} e^{{ - \left( {\lambda_{2} + \lambda_{phys} } \right)t}}$$

5 $$a_{4c} \left( t \right) = \frac{{\lambda_{1} }}{{\lambda_{2} + \lambda_{1} - \lambda_{3} }}\left( {e^{{ - \left( {\lambda_{3} + \lambda_{phys} } \right)t}} - e^{{ - \left( {\lambda_{1} + \lambda_{2} + \lambda_{phys} } \right)t}} } \right) + a_{1} e^{{ - \left( {\lambda_{1} + \lambda_{2} + \lambda_{phys} } \right)t}}$$

6 $$a_{5a} \left( t \right) = a_{1} e^{{ - \left( {\lambda_{1} + \lambda_{phys} } \right)t}} + a_{2} e^{{ - \left( {\lambda_{2} + \lambda_{phys} } \right)t}} - \left( {a_{1} + a_{2} } \right) e^{{ - \left( {\lambda_{3} + \lambda_{phys} } \right)t}}$$

7 $$a_{5b} \left( t \right) = \left( {a_{1} + a_{2} } \right) e^{{ - \left( {\lambda_{1} + \lambda_{phys} } \right)t}} - a_{1} e^{{ - \left( {\lambda_{2} + \lambda_{phys} } \right)t}} - a_{2} e^{{ - \left( {\lambda_{3} + \lambda_{phys} } \right)t}}$$

8 $$a_{6a} \left( t \right) = a_{1} e^{{ - \left( {\lambda_{1} + \lambda_{phys} } \right)t}} + a_{2} e^{{ - \left( {\lambda_{2} + \lambda_{phys} } \right)t}} + a_{3} e^{{ - \left( {\lambda_{phys} } \right)t}} - \left( {a_{1} + a_{2} + a_{3} } \right)e^{{ - \left( {\lambda_{3} + \lambda_{phys} } \right)t}}$$

9 $$a_{6b} \left( t \right) = a_{1} e^{{ - \left( {\lambda_{1} + \lambda_{phys} } \right)t}} + a_{2} e^{{ - \left( {\lambda_{phys} } \right)t}} - a_{3} e^{{ - \left( {\lambda_{2} + \lambda_{phys} } \right)t}} - \left( {a_{1} + a_{2} - a_{3} } \right)e^{{ - \left( {\lambda_{3} + \lambda_{phys} } \right)t}}$$

where ${a}_{px}(t)$ is a SOEF with $p$ estimated parameters and index $x$ for identification, the ${a}_{index}$ are the initial amplitudes of the respective exponential terms with values $\ge 0$. ${a}_{index}$ corresponds to the activity in the thyroid gland divided by the administered activity. ${\lambda }_{phys}$ is the physical decay constant of ^131^I (${\lambda }_{phys}=\text{ln}\left(2\right)/{T}_{1/2}$, with the ^131^I half-life ${T}_{1/2}$=8.022 days [4]), ${\lambda }_{index}$ are the biological clearance or uptake rates of the radiopharmaceutical with values $\ge 0$ [12, 13].

### Non-linear mixed-effects modeling

The NLME modelling (NLMEM) was used to estimate the fixed and random effects of the SOEF parameters in Eqs. (1)-(9), i.e. ${a}_{index}$ and ${\lambda }_{index}$. Fixed effects are the mean values of the estimated parameters in the population, while random effects show the variability of the estimated parameters as follows [14]:

10 $$P_{i} = TVP_{i} \times {\text{exp}}\left( {{{\varvec{\upeta}}}_{i} } \right)$$

11 $${{\varvec{\upeta}}}_{i} \sim N\left( {0,{{\varvec{\upomega}}}_{i}^{2} } \right)$$

Here ${P}_{i}$ is the estimated value of parameter $i$, $TV{P}_{i}$ is the fixed effect of parameter $i$ in the population, and ${{\varvec{\upeta}}}_{i}$ is a random number following a Gaussian distribution with mean zero and variance ${{{\varvec{\upomega}}}_{i}}^{2}$ (random effect, inter-individual variability) [15–17].

The variability of the measured data (random effect, intra-individual variability) was modelled using a proportional error model as follows:

12 $$\frac{{a\left( t \right) - a_{px} \left( t \right)}}{{a_{px} \left( t \right)}} = {{\varvec{\upvarepsilon}}}_{i}$$

where $a(t)$ is the fraction of the administered ^131^I activity in the thyroid gland at time $t$, ${a}_{px}(t)$ is the SOEF value at time $t$, and ${{\varvec{\upvarepsilon}}}_{i}$ is the random number following a Gaussian distribution with mean zero and variance of **σ**^2^ (random effect, intra-individual variability).

### Study workflow

The parameters of the SOEF (Eqs. (1)–(9)) were fitted to the complete set of thyroid retention data (*N* = 364) using the NLMEM method. All fittings were performed in the NONMEM software (version 7.5.1; ICON Development Solution, Ellicott City, MD, USA).

The PBMS-NLME approach was used to identify the SOEF that best characterises the measured data [10]. The fittings were repeated 1000 times with randomly varied starting values, and the best fit was used for the subsequent analysis. The goodness-of-fit was tested with the following criteria: visual inspection of the fitted curves is acceptable, the relative standard errors (RSEs) of the fitted parameters < 0.3 for fixed effects and < 0.5 for random effects (inter- and intra-individual variability) (“precise” criteria based on ref. [18] page 137), and the maximum absolute value of the off-diagonal elements of the correlation matrix < 0.8 (Table 1 in ref. [3]). The SOEF that successfully passed the goodness-of-fit test were subjected to model selection using the Akaike Information Criterion. The function with the highest Akaike weight was selected as the fit function most supported by the data [3, 10, 19]. The Akaike weights [3, 10, 19] of the fitted SOE functions were calculated as follows:

13 $$AICc = - 2ln\left( {\hat{L}} \right) + 2K + \frac{{2K\left( {K + 1} \right)}}{N - K - 1}$$

14 $$\Delta_{px} = AICc_{px} - AICc_{min}$$

15 $$w_{{AICc_{px} }} = e^{{\frac{{ - \Delta_{px} }}{2}}} /\sum\limits_{j \in F} {e^{{\frac{{ - \Delta_{j} }}{2}}} }$$

where $AICc$ is the corrected Akaike Information Criterion value, $\widehat{L}$ is the obtained minimum objective function value, K is the number of fitting parameters, ${AICc}_{px}$ is the $AICc$ of SOEF $px$, ${AICc}_{min}$ is the lowest $AICc$ value of all the investigated SOEF, ${\Delta }_{px}$ is the difference of the ${AICc}_{px}$ and ${AICc}_{min}$, $F$ is the set of all indices $px$ for which the SOEF ${a}_{px}(t)$ successfully passed the goodness-of-fit test and ${w}_{{AICc}_{px}}$ is the Akaike weight of function ${a}_{px}(t)$.

**Table 1:** Goodness of fits and Akaike weights for the investigated methods and functions

Method	Function name	K^a^	Relative standard error RSE of fixed effect (max)^b^	Relative standard error RSE of random effect (max)	Max absolute off-diagonal correlation matrix	OF (AICc)^c^	Akaike weight (%)^d^
1	${a}_{3a}$	7	0.41	0.23	0.79	− 1623 (− 1609)	-^f^
2	${a}_{3b}$^e^	7	0.44	0.22	0.77	− 1654 (− 1639)	-^f^
3	${a}_{4a}$	9	1.45	0.78	0.97	− 1622 (− 1603)	-^f^
4	${a}_{4b}$	9	0.14	1.01	0.69	− 1676 (− 1657)	-^f^
5	${a}_{4c}$	9	0.15	0.28	0.51	− 1836 (− 1818)	100
6	${a}_{5a}$	11	4.85	1.47	0.70	− 1622 (− 1599)	-^f^
7	${a}_{5b}$	11	0.25	0.43	0.43	− 1810 (− 1787)	2.03 × 10^–5^
8	${a}_{6a}$	13	0.37	1.89	0.71	− 1608 (− 1581)	-^f^
9	${a}_{6b}$	13	1.35	0.33	0.62	− 1785 (− 1758)	-^f^
10	${a}_{3b}$^g^	219^ h^	54.10	–	0.93	− 1456 (− 349)	< exp(-734)

The total number of measured data N included in this analysis is *N* = 364

^a^The number of parameters of the NLMEM for the corresponding SOEF (K is used in Eq. 13)

^b^The maximum value of the RSEs of the fit parameters

^c^the objective function (OF) and the Akaike information criterion corrected AICc from each NLMEM fitting

^d^The Akaike weight shows the likelihood that a function that meets the goodness-of-fit criteria (Sect. “Study workflow”) best describes the data

^e^SOE function ${a}_{3b}$ from the EANM SOP was fitted within the NLMEM framework

^f^Akaike weight was not calculated for SOEFs, which did not pass the goodness-of-fit test (Sect. “Study workflow”)

^g^SOEF ${a}_{3b}$ from the EANM SOP was fitted within the individual fitting framework as suggested in the literature [1]

^h^All biokinetic data from all patients were included in the analysis. The fitting was done individually, i.e. 3 parameters per patient for 73 patients. Although method 10 violates the goodness-of-fit criteria for at least one RSE value, the method was included as it is the standard method for thyroid dosimetry [1]

Internal model validation was performed to evaluate the fit of the function most supported by the data. This included assessments using basic goodness-of-fit plots, visual predictive checks (VPCs), normalised prediction distribution errors (NPDEs), and evaluation of parameter precision through the sampling importance resampling (SIR) approach [18]. To assess the impact of the selected residual error model, NLMEM fitting was performed using various residual error models, including additive, combined, and exponential models. The relative deviation of TIAs derived from these models was compared to that obtained from the proportional error model.

All SOEF ${a}_{px}(t)$ that passed the goodness-of-fit test were compared to the PBMS-NLMEM best-supported model by calculating relative deviations (RDs), root-mean-square errors (RMSEs), and mean absolute percentage errors (MAPE) of the TIAs. For each individual j, the relative deviation ${RD}_{px,j}$ was calculated for SOEF ${a}_{px}(t)$ as:

16 $$RD_{px,j} = \frac{{TIA_{px,j} - TIA_{PBMS - NLME, j} }}{{TIA_{PBMS - NLME, j} }},$$

and the root-mean-square error ${RMSE}_{px}$ and the mean absolute percentage error ${MAPE}_{px}$ over all patients as:

17 $$RMSE_{px} = \sqrt {\left( {SD RD_{px,j} } \right)^{2} + \left( {Mean RD_{px,j} } \right)^{2} }$$

18 $$MAPE_{px} = \frac{1}{n}\mathop \sum \limits_{j}^{n} \left| {RD_{px,j} } \right|$$

where $Mean {RD}_{px,j}$ and $SD {RD}_{px,j}$ are the mean and standard deviation of all ${RD}_{px,j}$, respectively. To assess reproducibility, the PBMS-NLME method was also implemented using MATLAB software version R2020 (https://www.mathworks.com/help/stats/nlmefitsa.html). The results were considered reproducible if: (1) the best SOEF from PBMS-NLME method identified by NONMEM and MATLAB software was the same, and (2) the differences in the estimated fixed and random effects parameters between NONMEM and MATLAB were less than 1%.

## Results

NLMEM fitting with SOEF ${a}_{3a}$*, *${a}_{3b}$*,* ${a}_{4a}, {a}_{4b}$, ${a}_{5a}$, ${a}_{6a}$, ${a}_{6b}$ did not pass the goodness-of-fit test, i.e. the fitting failed based on the visual inspection of the fitted curves and/or the RSEs of the estimated fixed effect < 0.3 and random effects (inter- and intra-individual variability) parameters < 0.5 (Table 1). Two functions passed the goodness-of-fit test, i.e. ${a}_{4c}$ and ${a}_{5b}$. SOEF ${a}_{4c}$ was identified as the function most supported by the biokinetic data in PBMS-NLME with an Akaike weight of approximately 100% (Table 1). Table 2 shows the estimated parameters from the NLMEM fitting with SOEF ${a}_{4c}$.

**Table 2:** Parameters estimated from all-time-point fitting using the best SOEF PBMS-NLME (${a}_{4c}$)

	Model Parameters (unit)	Estimate	%RSE^e^	95% CI^e^
Fixed effect	${a}_{1}$	7.1 × 10^–2^	10	[5.8 × 10^–2^, 8.6 × 10^–2^]
	${\lambda }_{1}$^a^ (1/h)	7.9 × 10^–2^	10	[6.5 × 10^–2^, 9.8 × 10^–2^]
	${\lambda }_{2}$^b^ (1/h)	6.7 × 10^–2^	7	[5.8 × 10^–2^, 7.6 × 10^–2^]
	${\lambda }_{3}$^c^ (1/h)	1.2 × 10^–3^	15	[8.6 × 10^–4^, 1.6 × 10^–3^]
Inter-individual variability^d^	${a}_{1}$	0.79	13	[0.56, 1.1]
	${\lambda }_{1}$(1/h)	1.1	9	[0.89, 1.47]
	${\lambda }_{2}$(1/h)	0.51	12	[0.41, 0.66]
	${\lambda }_{3}$(1/h)	1.0	13	[0.72, 1.64]
Intra-individual variability		4.8 × 10^–2^	6	[4.2 × 10^–2^, 5.4 × 10^–2^]

^a^Biological rate ${\lambda }_{1}$ corresponds to a half-life of T_1/2_ = 8.7 h

^b^Biological rate ${\lambda }_{2}$ corresponds to a half-life of T_1/2_ = 10.4 h

^c^Biological rate ${\lambda }_{3}$ corresponds to a half-life of T_1/2_ = 555 h

^d^The inter-individual variability is presented as the CV

^e^95% CI and %RSE values were calculated using the SIR approach

The basic goodness-of-fit plots, VPC, and NPDE demonstrate that the SOEF ${a}_{4c}$ model performs well in describing the observed ^131^I data (Supplemental File, Figure S1). The residual error models exhibited a minimal impact on the calculated TIAs with RDs when compared to the proportional residual error model, with observed differences of (-0.03 ± 3.96)%, (0.25 ± 1.91)%, and (− 0.13 ± 0.11)% for the additive, combined, and exponential residual error models, respectively. These findings indicate that the choice of residual error model had a negligible effect on the calculated TIA in our datasets. Consequently, the proportional residual error model was selected for the subsequent analysis in this study.

**Fig. 1:**
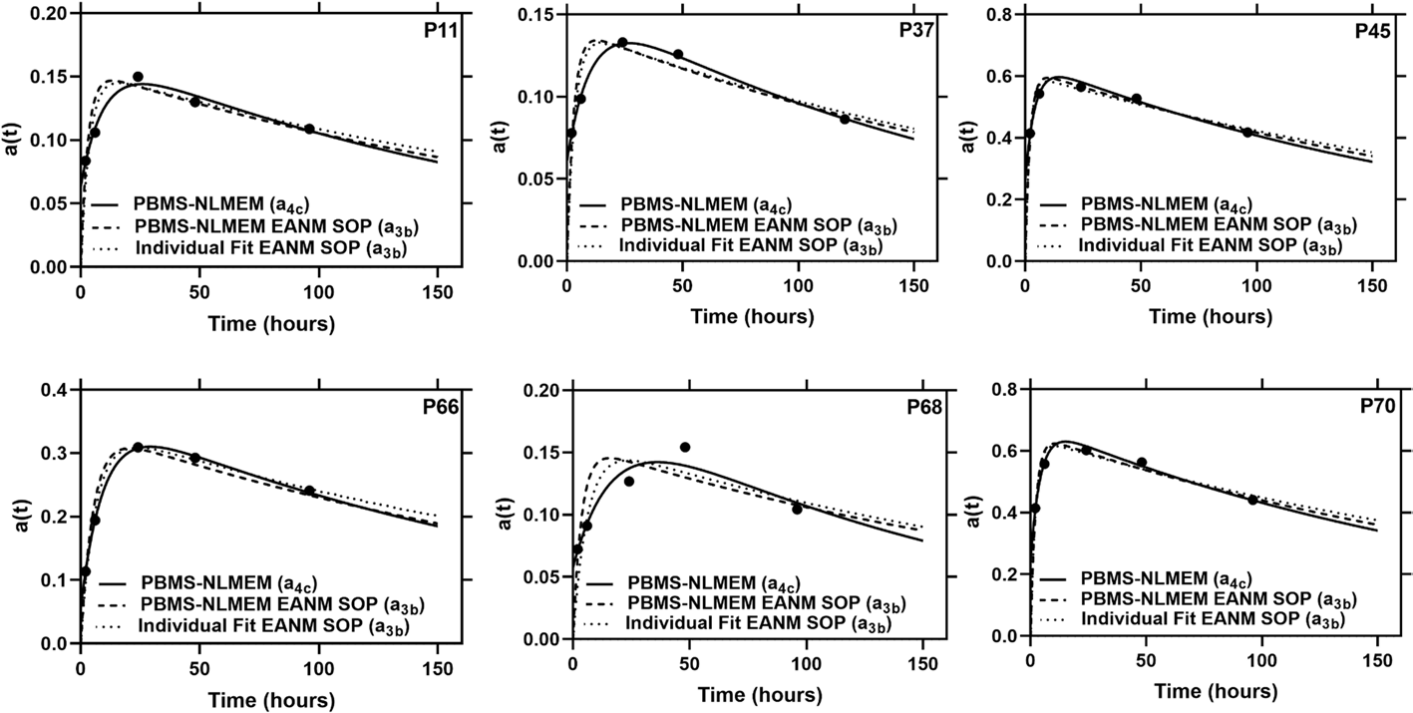
Measured fraction of the administered activity in the thyroid gland a(t) as a function of time t and fit curves obtained from the PBMS-NLME (${a}_{4c}$), PBMS-NLME EANM SOP (${a}_{3b}$), and Individual Fit EANM SOP (${a}_{3b}$) method. The curves for the 6 patients with the highest RD for the Individual Fit EANM SOP are shown. The fitted curves of all patients are presented in the supplemental file Figure S2

**Fig. 2:**
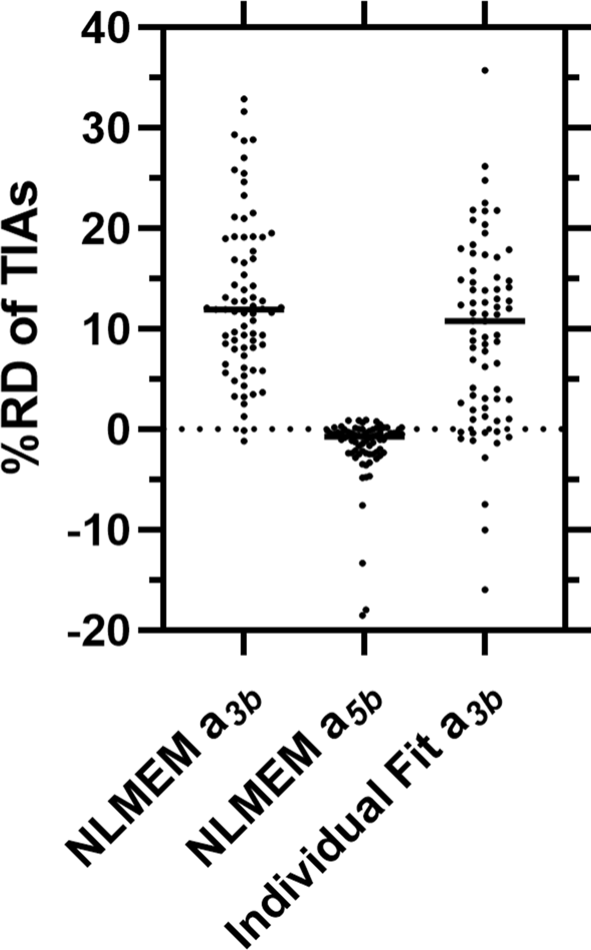
%RDs of TIAs obtained from the NLMEM (${a}_{3b}$), NLMEM (${a}_{5b}$), and Individual Fit (${a}_{3b}$) method with TIAs obtained from the PBMS-NLMEM (${a}_{4c}$) as the reference

**Fig. 3:**
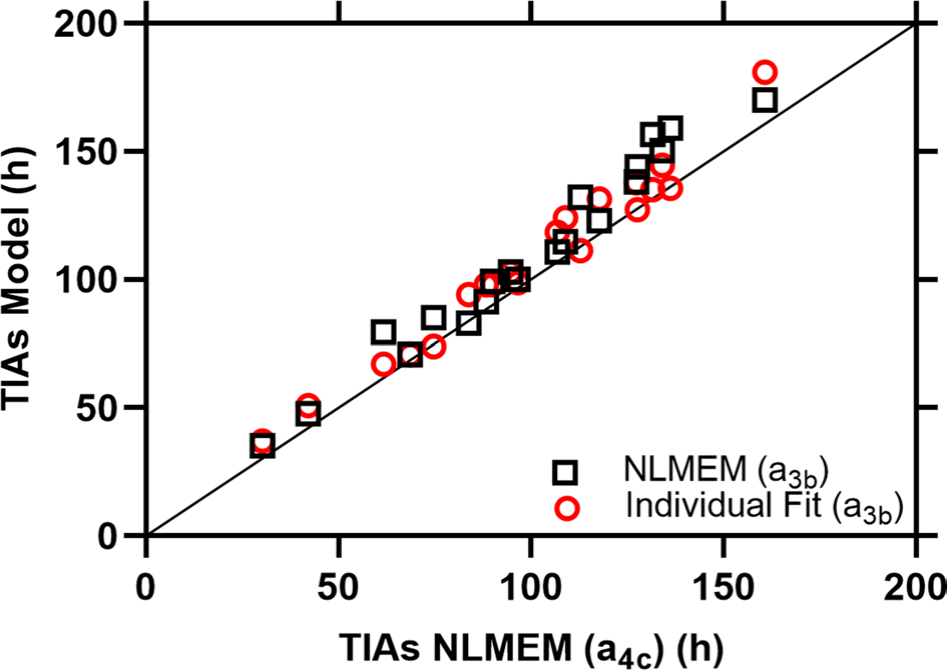
Comparison of the TIAs obtained from the NLMEM and Individual Fit EANM SOP (${a}_{3b}$) with the NLMEM best fit SOEF (${a}_{4c}$) for the 20 patients with measurements at *t* = 2, 6, 24, 48, and 120 h post oral administration as suggested by the EANM SOP [1]

Figure 1 compares the curves obtained from the best SOEF from PBMS-NLMEM ${a}_{4c}$, individual fitting with the EANM SOP SOEF ${a}_{3b}$, NLMEM fitting with the EANM SOP function ${a}_{3b}$ in 6 patients with highest RD for individual fit EANM SOP (Supplemental file Figure S2 shows all patients). The best SOEF from PBMS-NLME ${a}_{4c}$ shows a good performance in determining the biokinetic data in all patients (Fig. 1). Visual inspection of the fitted graphs in Figs. 1 and S2 shows that the best SOEF from the PBMS-NLME method ${a}_{4c}$ has a better or equivalent performance as the curves from the individual fitting with SOEF of the EANM SOP ${a}_{3b}$, and NLMEM fitting with the EANM SOP function ${a}_{3b}$. Figure 2 and Table 3 show the RD of the TIAs calculated with NLMEM EANM SOP ${a}_{3b}$, NLMEM ${a}_{5b}$ and individual fitting EANM SOP ${a}_{3b}$ to the TIAs calculated using the best SOEF from PBMS-NLME ${a}_{4c}$. Individual fitting with EANM SOP ${a}_{3b}$ outperforms PBMS-NLME ${a}_{3b}$ based on the RMSE and MAPE values (Table 3). Figure 3 shows the individual TIAs of function ${a}_{4c}$, and function ${a}_{3b}$ with individual and NLMEM fitting for 20 patients with measurement data at *t* = 2, 6, 24, 48, and 120 h post oral administration. TIAs calculated from both SOEF ${a}_{3b}$ with individual and NLMEM fitting were relatively higher than TIAs of NLMEM function ${a}_{4c}$ with relative deviations up to 23% and 29%, respectively (Fig. 3). The PBMS-NLME results were reproducible, identifying the same SOEF ${a}_{4c}$ as the best function from PBMS-NLME method in both NONMEM and MATLAB software, with less than a 1% difference in the estimated fixed and random effects parameters between the two platforms (data not shown).

**Table 3:** The RD, RMSEs, and MAPE of different methods for models passing the goodness-of-fit test in comparison to the PBMS-NLME (${a}_{4c}$) reference for 20 patients with measurements at *t* = 2, 6, 24, 48, and 120 h post oral administration as suggested by the EANM SOP [1]

Methods	RD (%)	RMSE of the RDs (%)		MAPE (%)
	Mean (SD)	Median [min, max]		
PBMS-NLME EANM SOP ${a}_{3b}$	10.3 (7.2)	9.4 [− 1.2, 28.7]	12.5	10.4
PBMS-NLME ${a}_{5b}$	− 1.1 (1.5)	− 0.4 [− 4.8, 0.9]	1.8	1.2
Individual Fit EANM SOP ${a}_{3b}$	8.1 (6.8)	8.6 [− 1.4, 22.5]	10.6	8.4

## Discussion

The accurate determination of individual TIAs is highly desirable in MRT. In 2013, the EANM introduced an SOP for pre-therapeutic dosimetry of radioiodine therapy, employing a bi-exponential function for three or more uptake assessments and approximations for two or only one measurement [4]. The underlying theory assumes first-order kinetics with instantaneous transfer of the administered activity into the blood pool and mono-exponential decay from the blood pool. However, absorption into the bloodstream from the stomach and intestines is slightly delayed, which means that the activity concentration in the blood immediately after application is lower than theoretically expected. On the other hand, a much faster than expected early increase in activity in the target tissue may occur due to the first-pass effect prior to equilibration, which can contribute a few percent of the absorbed dose in benign thyroid diseases [20] and the fact that the volume of distribution immediately after absorption into the blood is significantly lower than at later times [21], resulting in a faster than a mono-exponential decrease in activity concentration in the blood in the first hours after the administration. Furthermore, the fraction of blood pool activity in a field of view above the neck tends to be underestimated by a measurement on the thigh. Thus, the retention in the target tissue is overestimated in early measurements.

Therefore, using the bi-exponential function and individual fitting method outlined in the EANM SOP may not universally apply, necessitating a statistical evaluation of its performance. Model selection is an essential and critical aspect of scientific data analysis [8, 11]. Recent evidence has demonstrated that the PBMS-NLME method outperforms individual-based model selection [10]. The PBMS-NLMEM method employs statistical analysis and objective criteria to determine the optimal SOEF in MRT dosimetry.

Based on the goodness-of-fit test and Akaike weight of approximately 100%, NLMEM fitting with SOEF ${a}_{4c}$ was identified as the function most supported by the data (Table 1). The SOEF ${a}_{4c}$ model exhibits strong performance in capturing the observed ^131^I data, as evidenced by the basic goodness-of-fit plots, VPC, and NPDE (Supplemental File, Figure S1). Consistent results were obtained using the PBMS-NLME method in MATLAB, where the SOEF ${a}_{4c}$ was identified as the optimal function, showing less than a 1% difference between the estimated fixed and random effect parameters from NONMEM and MATLAB software. SOEF ${a}_{4c}$ has one more parameter than the SOEF ${a}_{3b}$ of the EANM SOP, potentially resulting in more accurate TIAs. Identification of an SOEF with more exponential terms and parameters using the PBMS-NLMEM approach than the EANM SOP function is expected due to the oral and stomach uptake. Figure 1 illustrates how the selected fit function may lead to a considerable difference in the calculated TIAs: the fitted curves using function ${a}_{4c}$ appear more appropriate than the EANM SOP function ${a}_{3b}$ in describing the biokinetic data in several patients, e.g. in patients P2 and P73. Additionally, the RMSE value for EANM SOP function ${a}_{3b}$ with NLMEM and individual fitting was 12.5% and 10.6%, respectively, which is relatively high and might affect the calculation of the activity to administer in ^131^I therapy. While the SOEF ${a}_{4c}$ demonstrated acceptable performance in modelling the biokinetic data of ^131^I in our population, this function may not optimally represent the subpopulation of non-goitre patients, as this group was represented by only 2 out of 73 patients.

Care must be taken when using the EANM SOP function, especially when including an early data point with a high value. This could lead to an overestimation of the blood decay rate and an underestimation of the decay rate in the target tissue, resulting in an overestimated TIA. The distinction between the PBMS-NLME model’s best SOE function a_4c_ and the EANM SOP SOE function a_3b_ lies in considering the blood pool fraction during thyroid measurements. Apparently, the count rates from the activity in the blood pool are not identical in the neck and thigh measurements, and the subtraction of the thigh activity does not fully correct for the blood pool component in the neck measurement, necessitating the inclusion of parameter ${a}_{1}$ in the a_4c_ SOEF (Supp. File). The result shows that two exponentials suffice, as all SOEFs with 3 or 4 exponentials are less supported by the data. Thus, the Two-Compartment Model of the EANM SOP is sufficient to describe the data. The optimal SOEF model identified in this study is based on a physical model, i.e. the EANM SOP model with subtraction of measured thigh activity. Although the SOEF parameters could be different between centres due to the preparation of the patients, other centres can nevertheless adopt the SOEF model structure. Depending on the technical implementation of the measurements and the patient population, adjusted fixed effect values and random effect variances for the parameters might be adequate. However, the values obtained with our data should not be regarded as generally valid for all centres without verification.

Calculating the TIAs using individual fitting with EANM SOP ${a}_{3b}$ [1] requires at least four data points to adhere to the constraint of K_max_ (maximum number of fitted parameters) = N (number of data) − 1 of the individual fitting [22]. In contrast, the NLMEM fitting employs a population-based approach, where the ratio between the number of data points and the number of fitted parameters is higher than in individual fits [8, 17]. In this study, the ratios of the total number of data points to the estimated parameters are 364/9 (= 40.4) and 5/3 (= 1.67) for PBMS-NLME ${a}_{4c}$ with NLMEM population fitting and EANM SOP ${a}_{3b}$ with individual fitting, respectively. Since the ratio of the total number of data to the estimated parameters co-determines the quality of the fitting (for the case of a function passing the goodness-of-fit test), in addition to the Akaike weight comparison (Tab. 1) and the quality of the fitted curves (Supp. 1), it can be concluded that PBMS-NLME ${a}_{4c}$ with NLMEM population fitting outperforms the EANM SOP ${a}_{3b}$ with individual fitting.

In our study, all structural parameters demonstrated high inter-individual variability (Table 2). High inter-individual variability in the model can lead to unreliable results when using empirical Bayes estimates, particularly when sparse data is available for individual patients. One potential solution to mitigate this issue is the incorporation of covariates into the model. Population pharmacokinetic analysis with NLME provides some advantages, including identifying factors that affect pharmacokinetic parameters and explaining inter-individual variability through covariate modelling [23]. The optimal SOE model identified via the PBMS-NLME method can be employed for covariate modelling to improve the precision of individual pharmacokinetic estimations and therapeutic activity. A study analysing ^131^I kinetics in the patient population is underway.

Our study employed a strategic approach by starting with a comprehensive list of SOEFs, enabling us to explore the number of exponential terms needed for accurately structuring the compartmental model. This flexible methodology allowed us to test various configurations and determine the model best suited to the biokinetics of ^131^I. By doing so, we could ensure that the model selected was not only theoretically sound but also empirically validated through the data. In particular, it was shown that none of the investigated functions with more than 2 exponentials has a relevant probability of being a suitable model to describe the data. Thus, a two-compartmental model efficiently described the data; more compartments were unnecessary. It is important to emphasise that the best-performing function identified in our study aligns with a physical model. This alignment underscores the robustness of our approach and supports the validity of the model within the context of thyroid biokinetics. Moreover, by employing this strategy, we have enhanced the potential for broader applicability of our findings, allowing other researchers or clinical centres to adopt these parameters in their settings with greater confidence.

## Conclusions

An improved mathematical model (function ${a}_{4c}$) was determined for calculating TIAs in ^131^I therapy dosimetry for benign thyroid diseases based on the PBMS-NLME method. Notably, the performance of the function ${a}_{4c}$ surpasses that of individual fitting as outlined in EANM SOP ${a}_{3b}$. Therefore, it is recommended to adopt the PBMS-NLME approach for thyroid dosimetry.

## Supplementary Information

Additional file1 (DOCX 7558 KB)

## Data Availability

The used data are available from the corresponding author upon reasonable request.
