# Supplementary material for: Non-linear mixed-effects modelling and population-based model selection for 131I kinetics in benign thyroid disease
Source: EJNMMI Phys. 2025 Apr 8;12:37. doi: 10.1186/s40658-025-00735-6 (PMC11979076; doi:10.1186/s40658-025-00735-6)
Supplement: Supplementary file 1 — Additional file1 (DOCX 7558 KB) [file 40658_2025_735_MOESM1_ESM.docx]

**Supplemental File**

Non-Linear Mixed-Effects Modelling and Population-Based Model Selection for ^131^I Kinetics in Benign Thyroid Disease

Deni Hardiansyah^1$^, Ade Riana^1$^, Heribert Hänscheid^2^, Ambros J. Beer^3^, Michael Lassmann^2^ Gerhard Glatting^3,4*^

^1^Medical Physics and Biophysics, Physics Department, Faculty of Mathematics and Natural Sciences, Universitas Indonesia, Depok, Indonesia

^2^Department of Nuclear Medicine, University Hospital Würzburg, Würzburg, Germany

^3^Department of Nuclear Medicine, Ulm University, Ulm, Germany

^4^Medical Radiation Physics, Department of Nuclear Medicine, Ulm University, Ulm, Germany

^$^Equal contribution


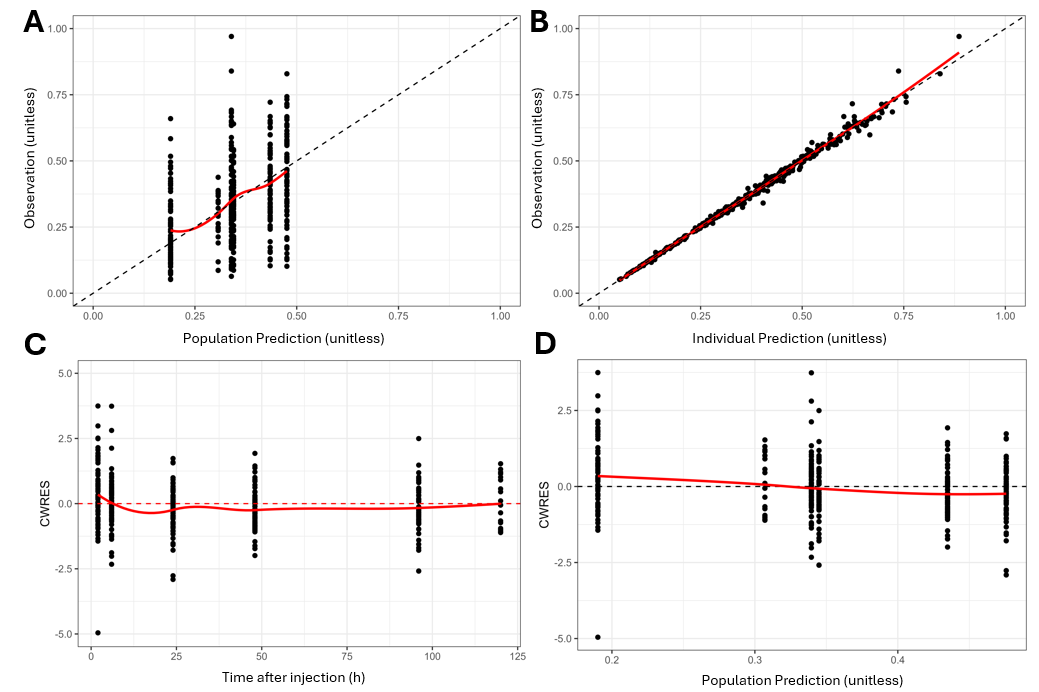


**
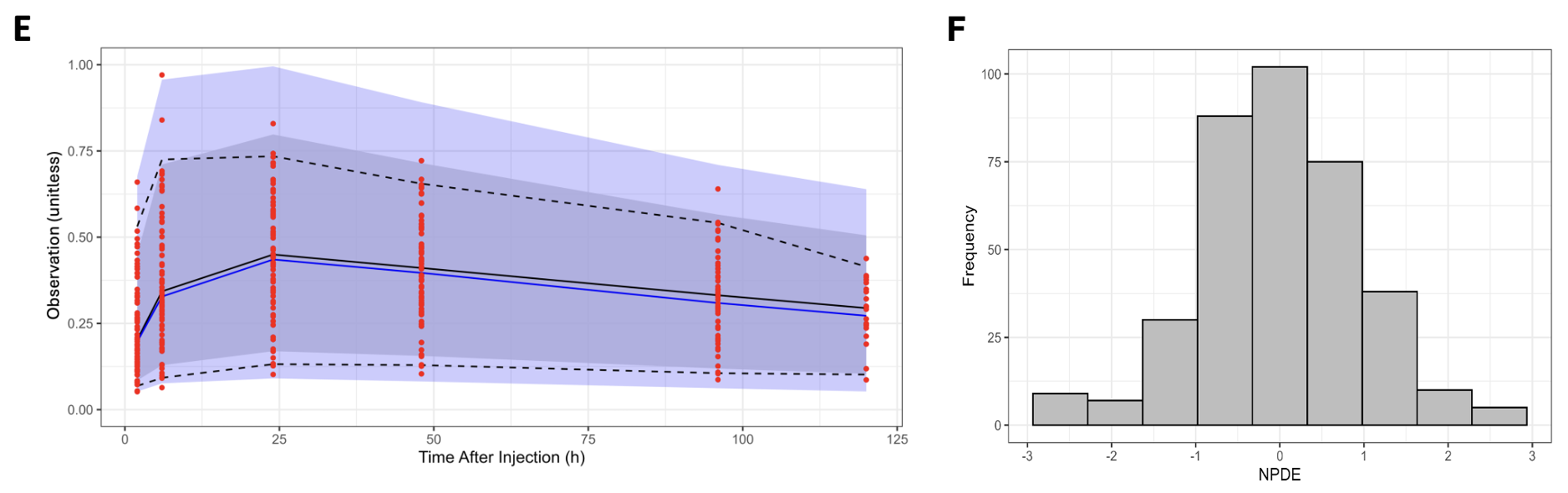
**

**Figure S1.** Goodness-of-fit plot assessment of the Sum-of-exponential function (SOEF) $a_{4c}$ [1]. A) Population prediction (PRED) versus observations, B) individual prediction (IPRED) versus observation, C) conditional weighted residual (CWRES) versus time after injection, D) CWRES versus population prediction, E) Virtual Predictive Check (VPC) plot based on 10,000 simulations, and E) normalised prediction distribution error (NPDE).


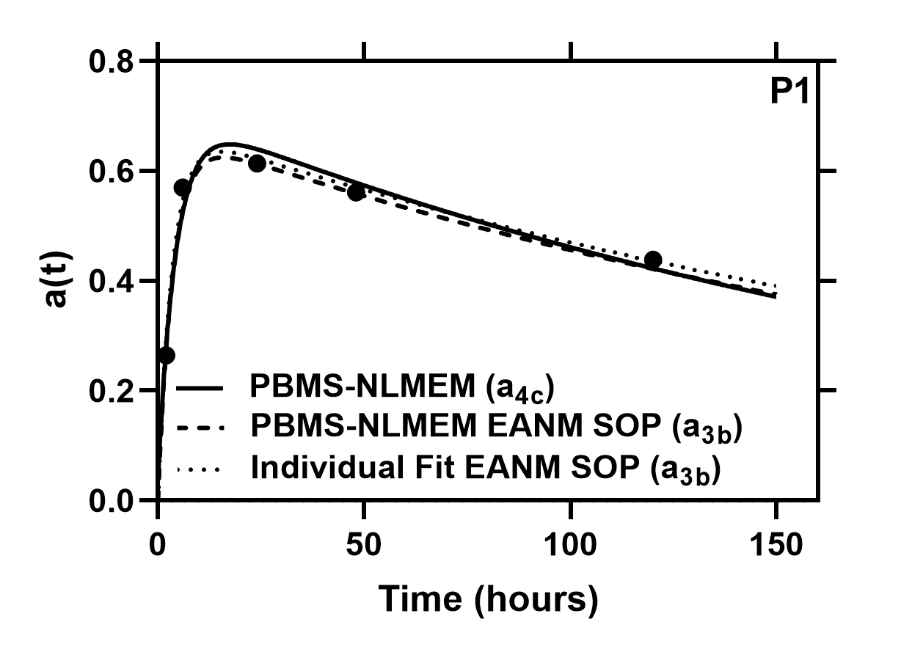

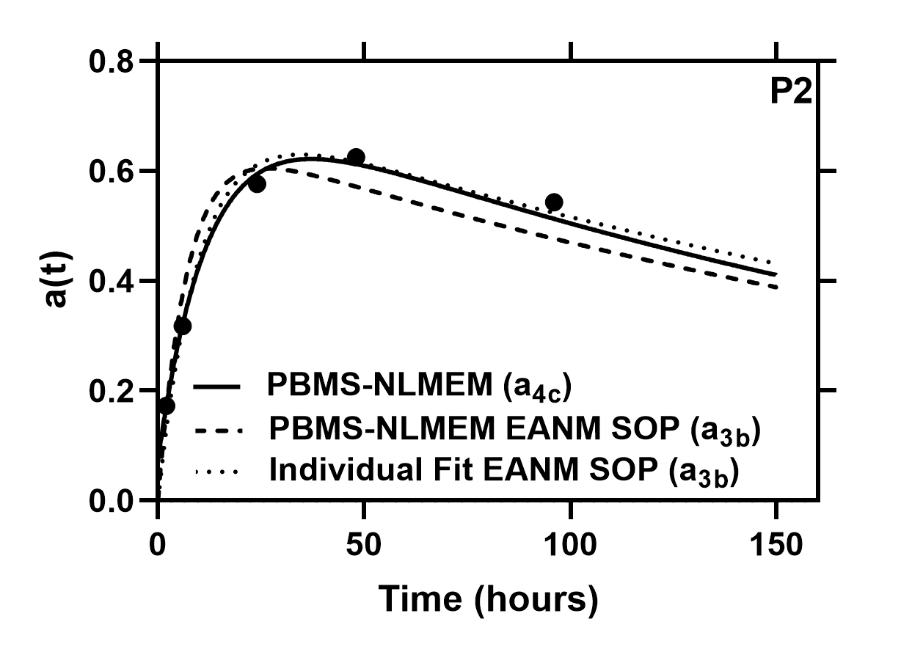

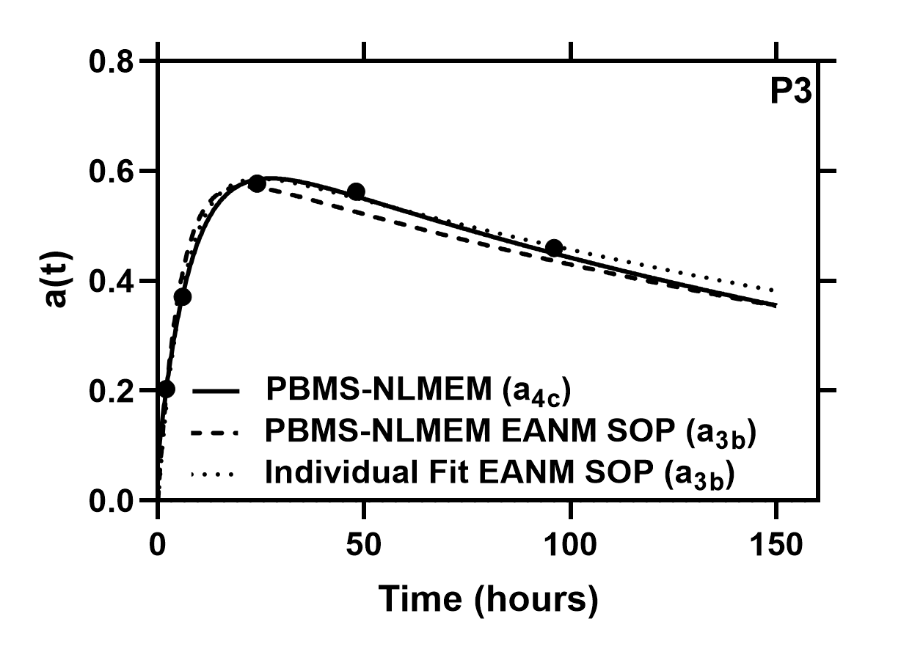

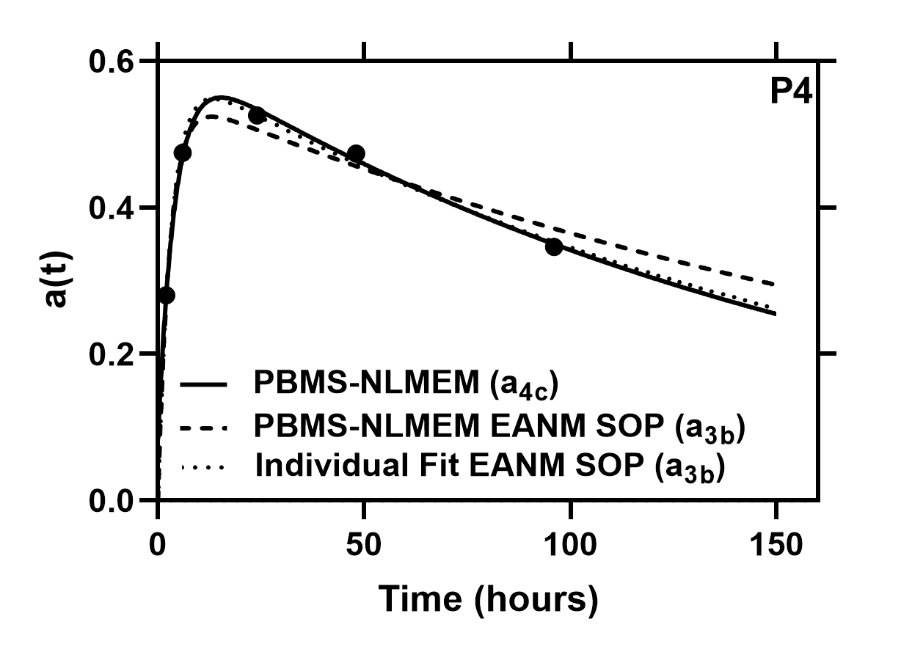

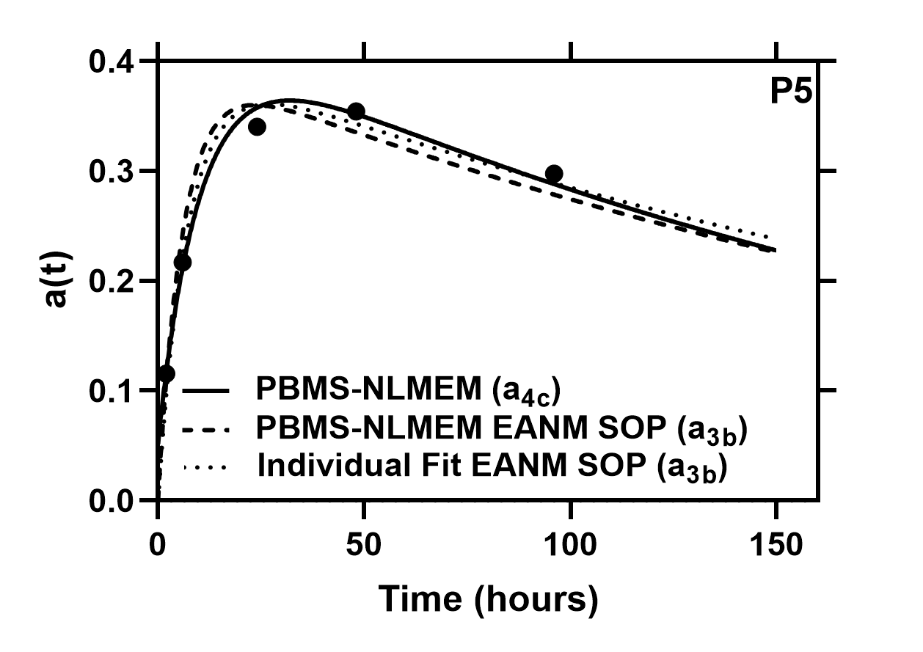

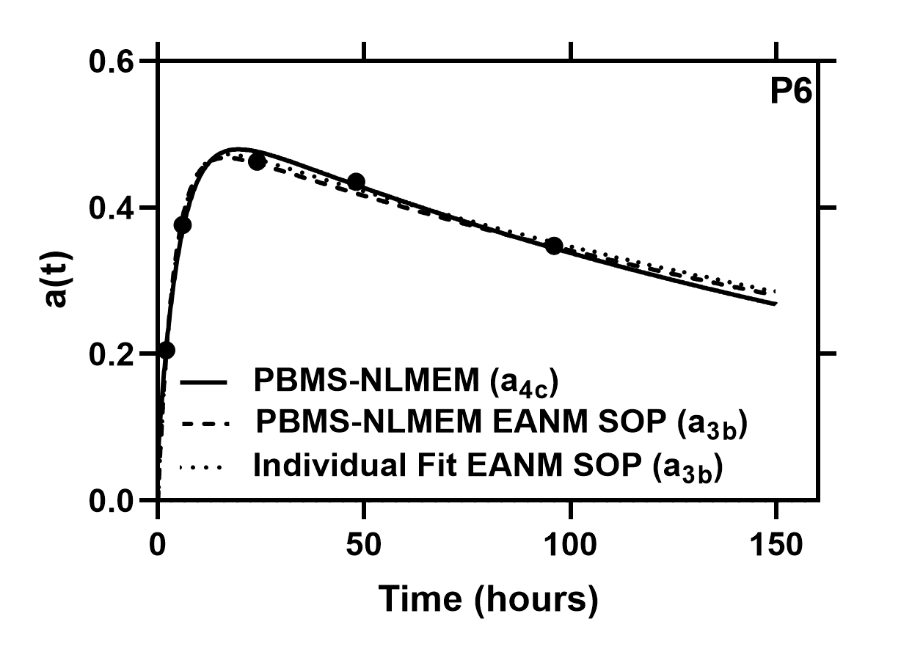

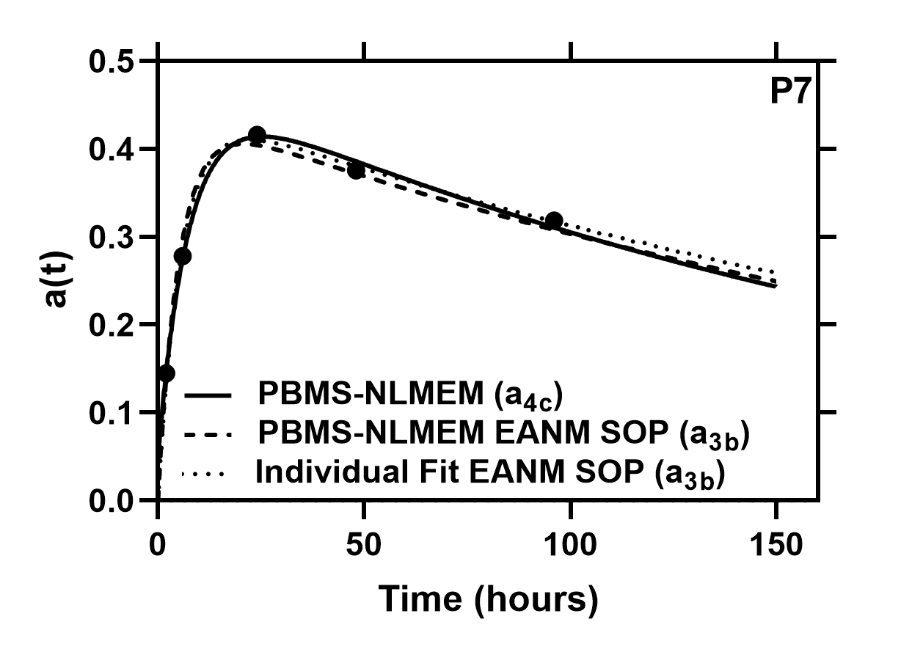

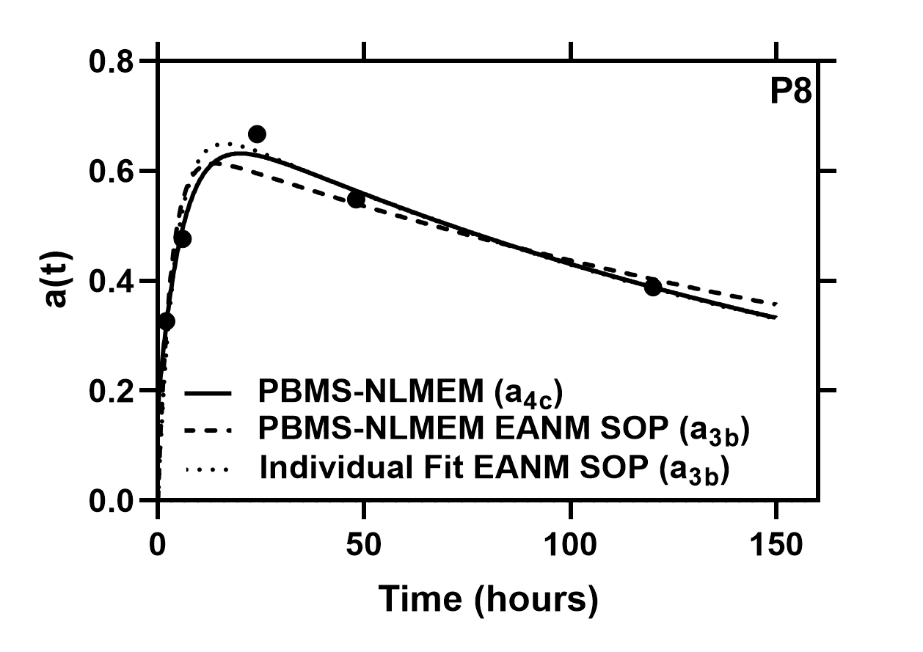

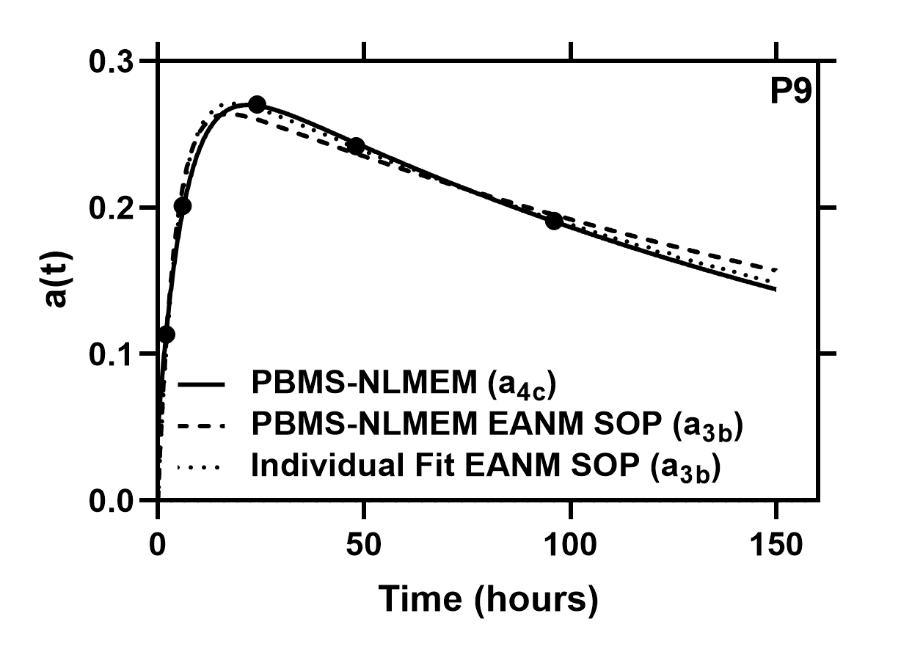

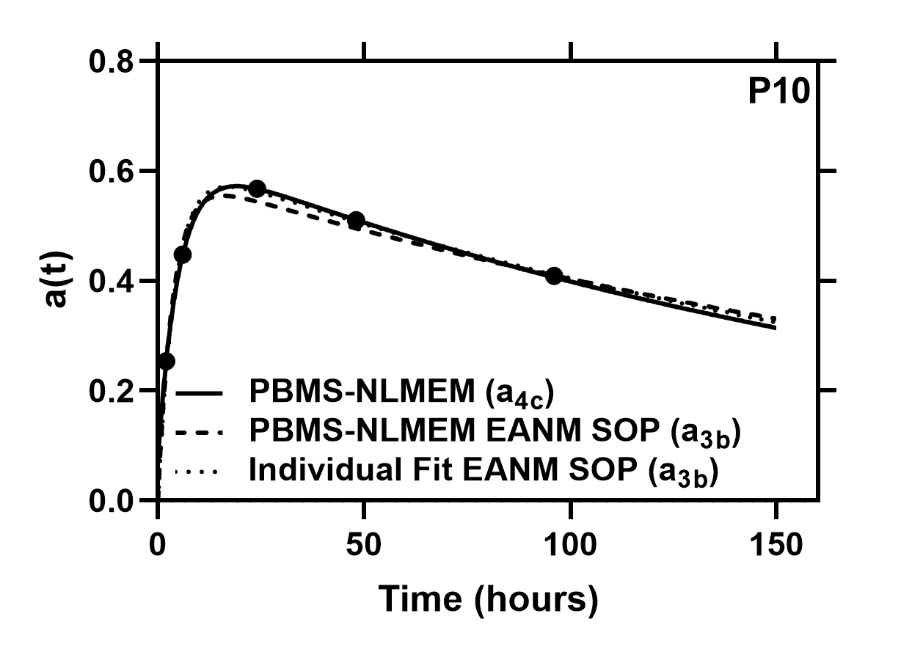

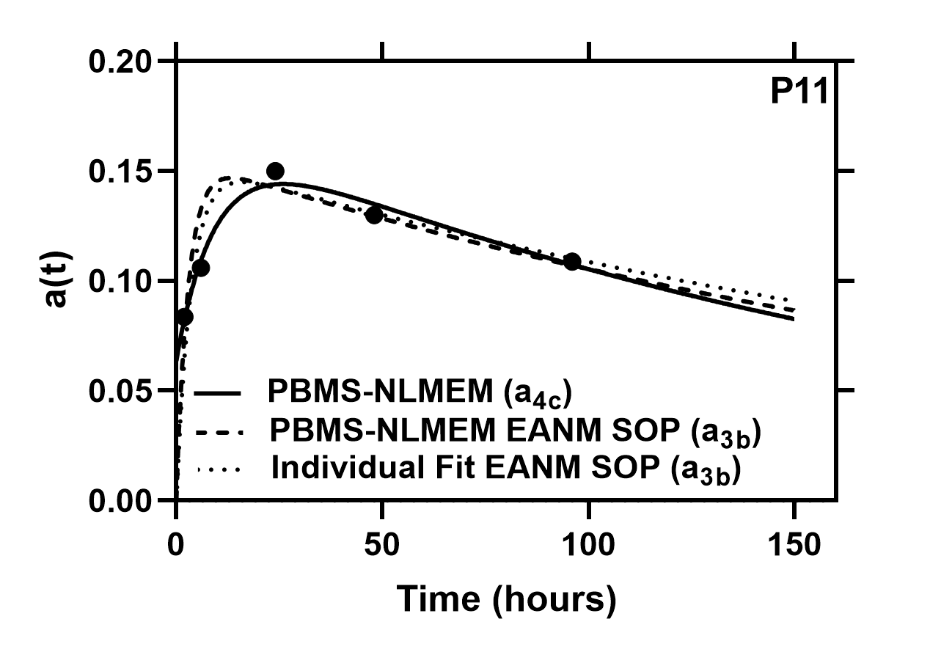

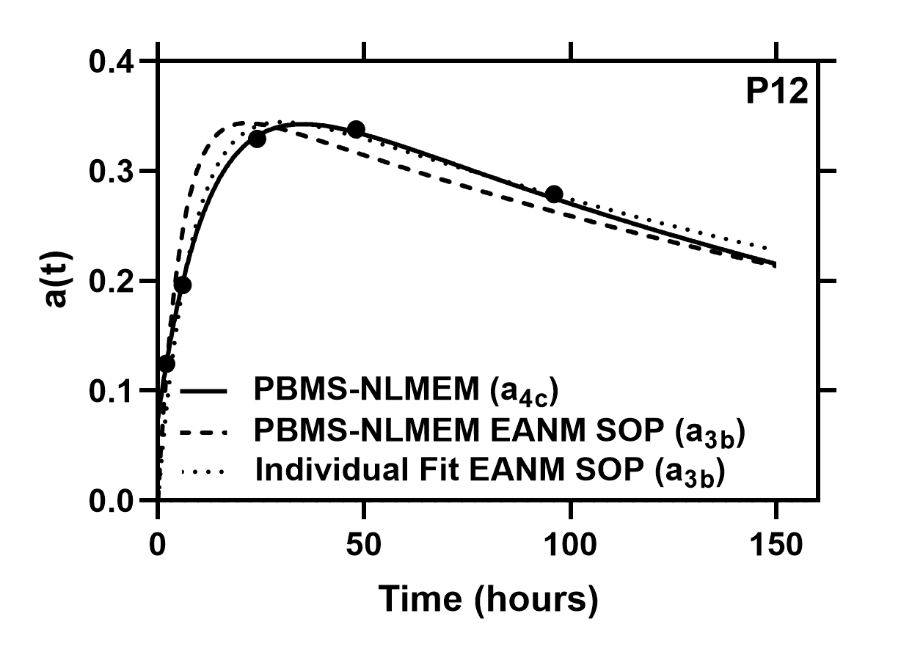

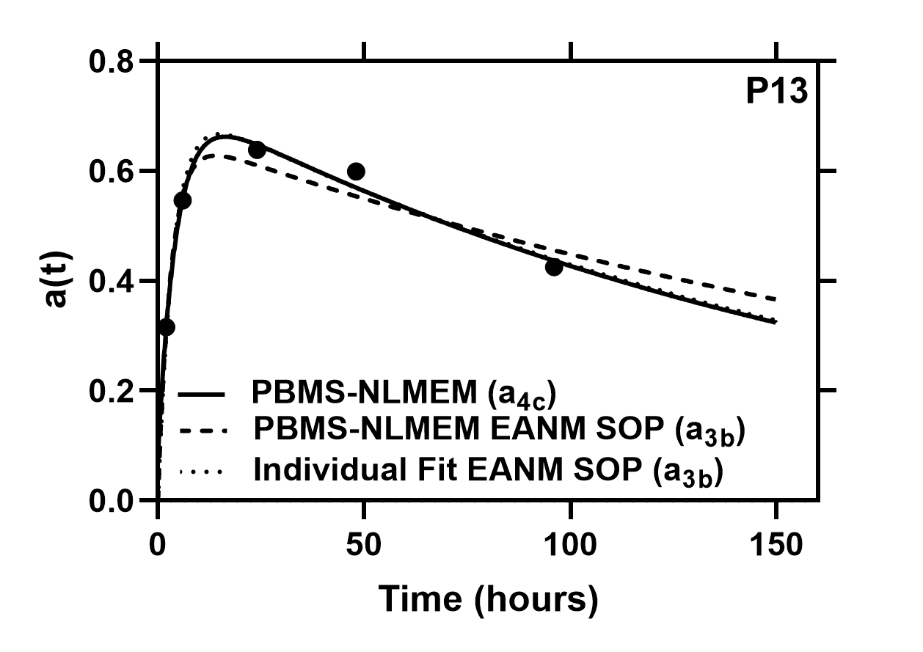

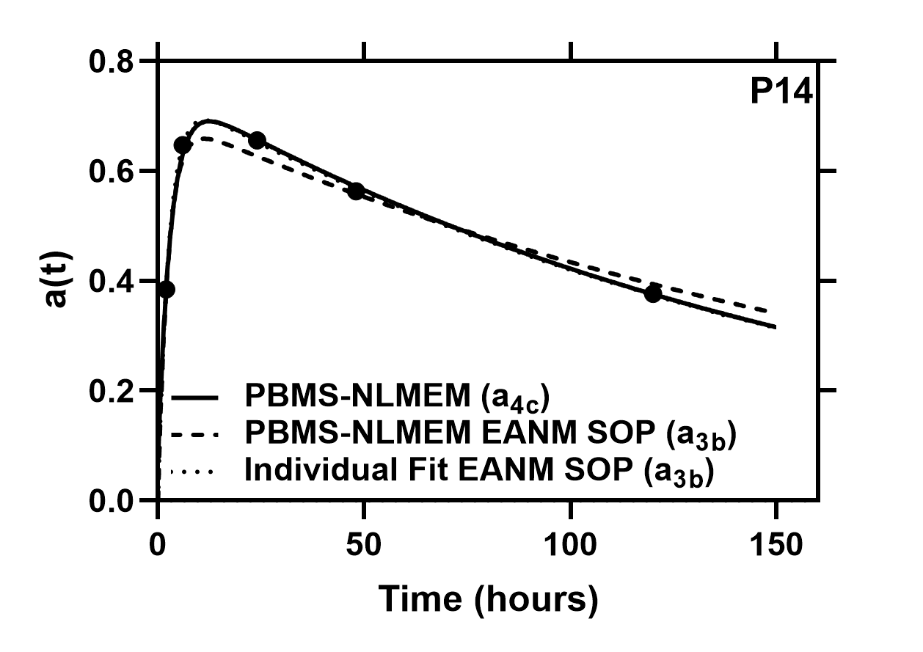

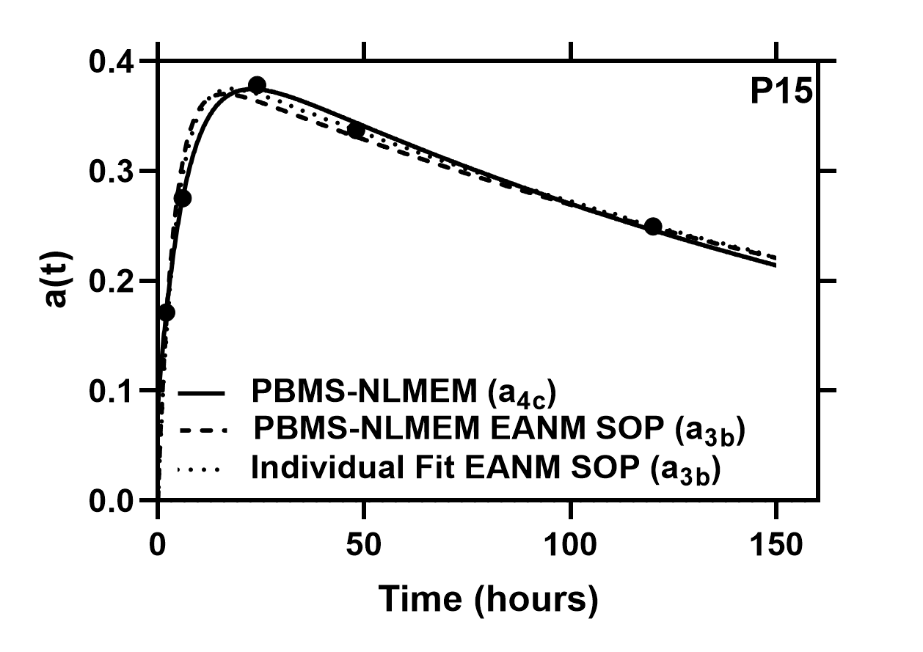

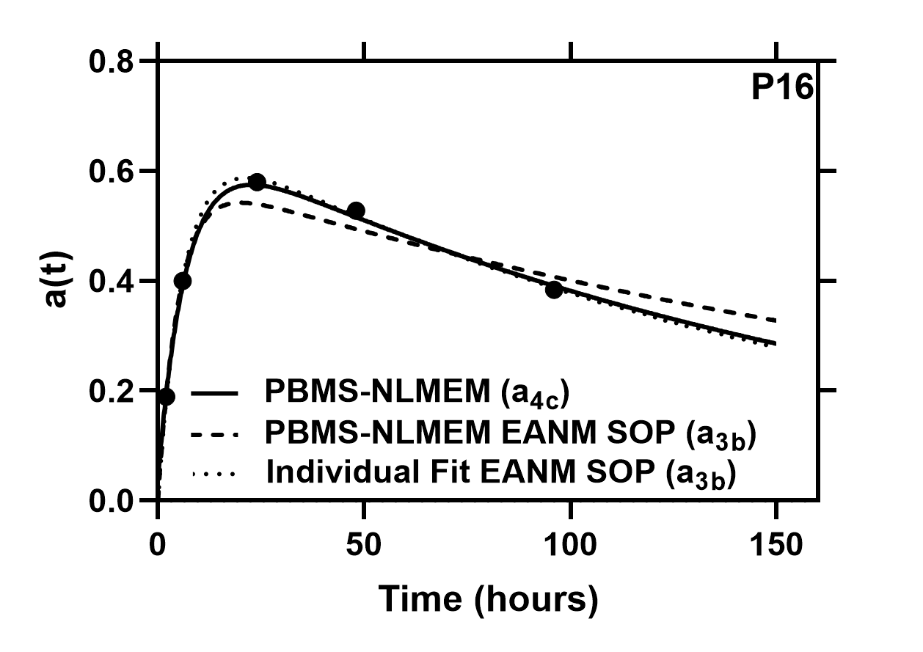

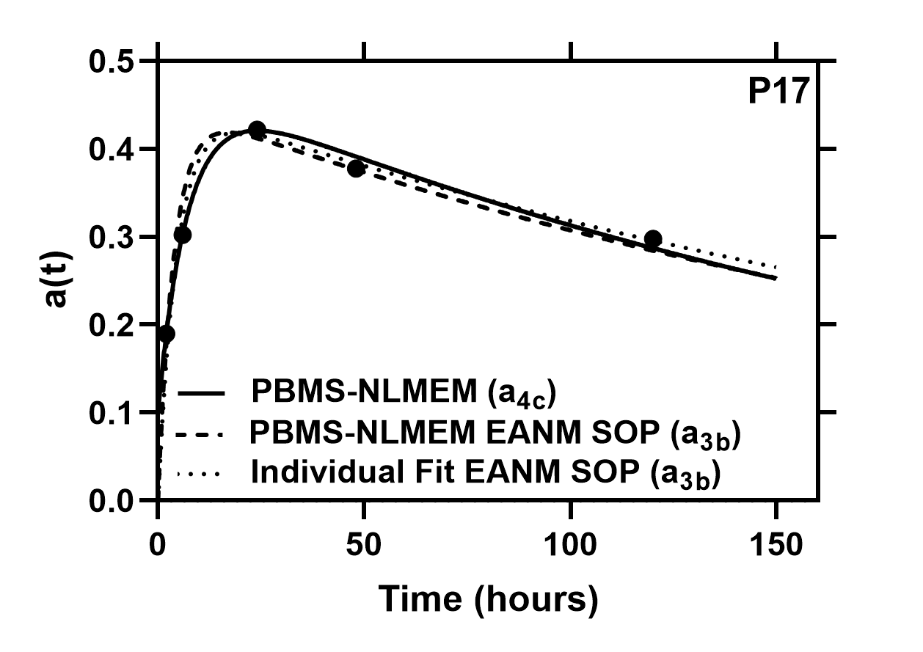

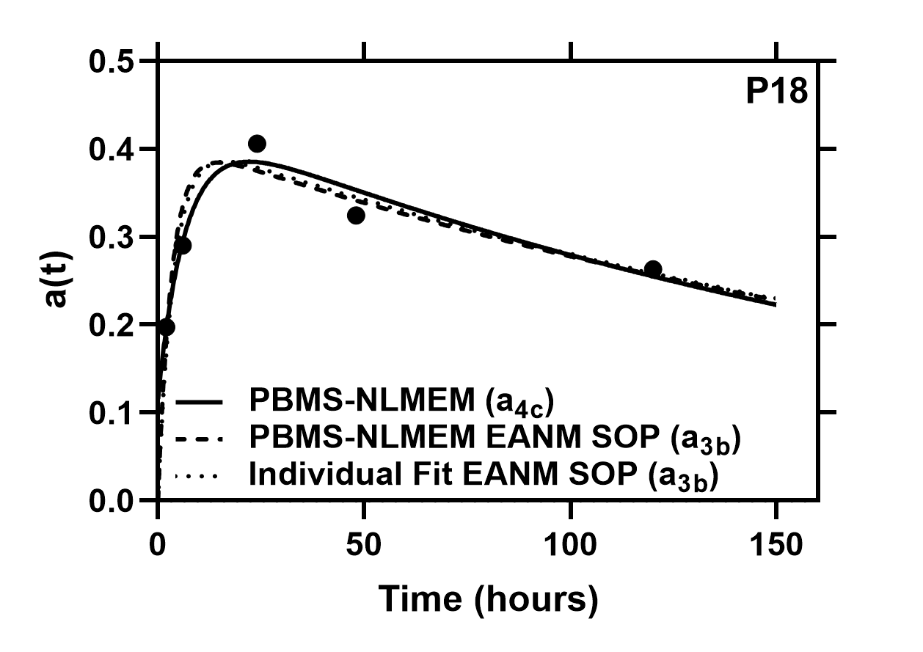

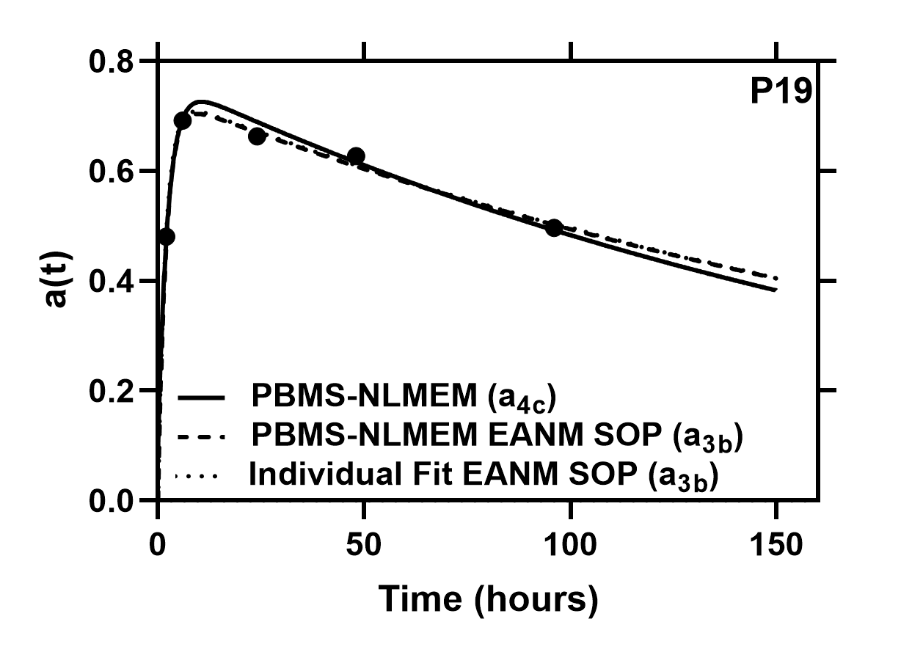

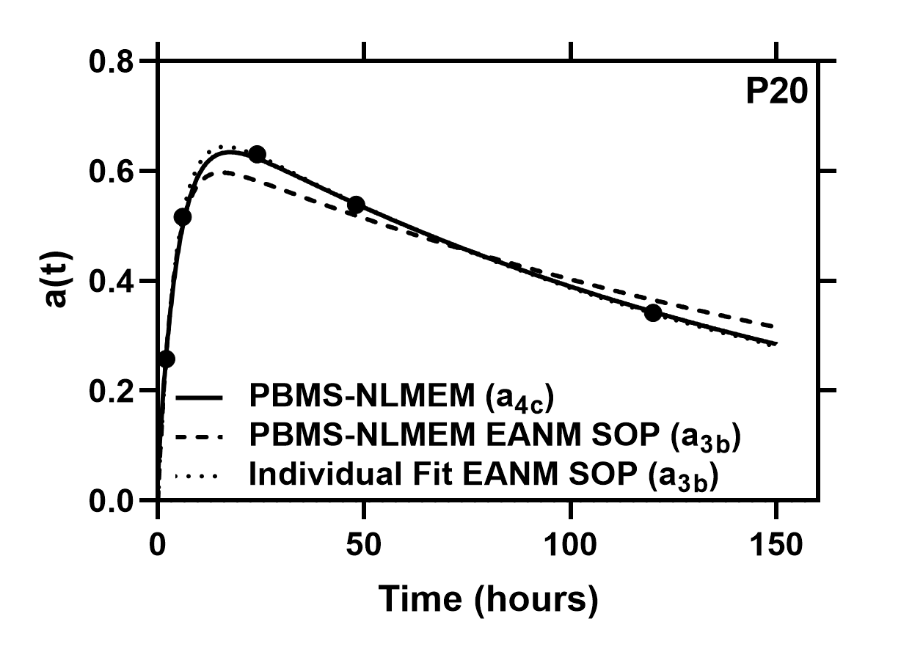

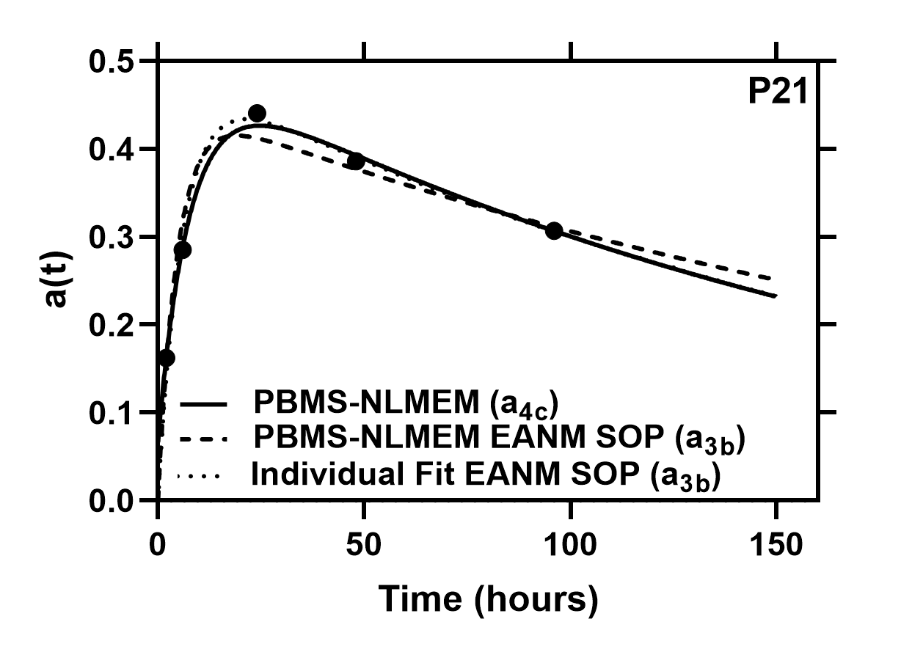

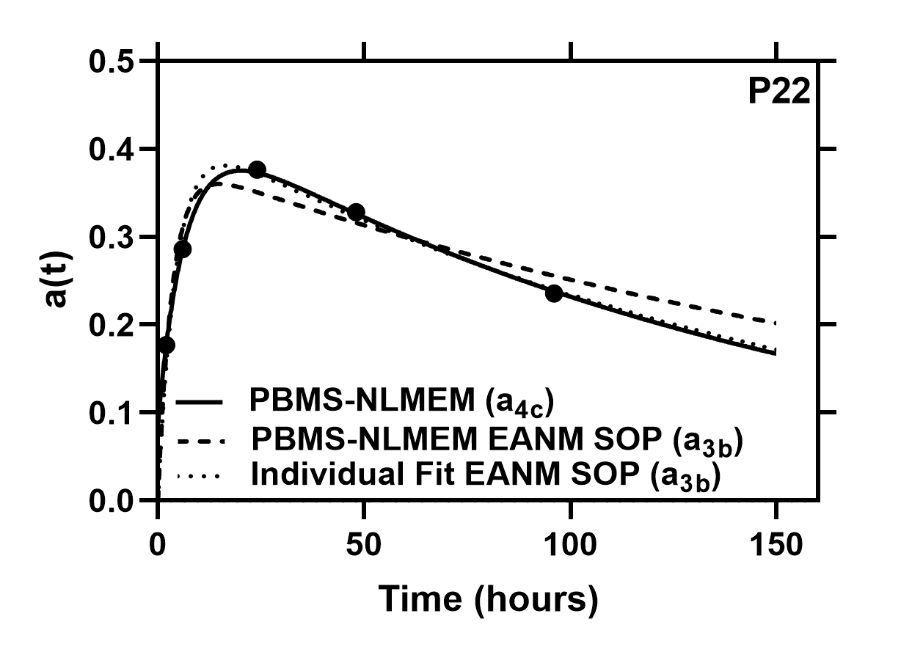

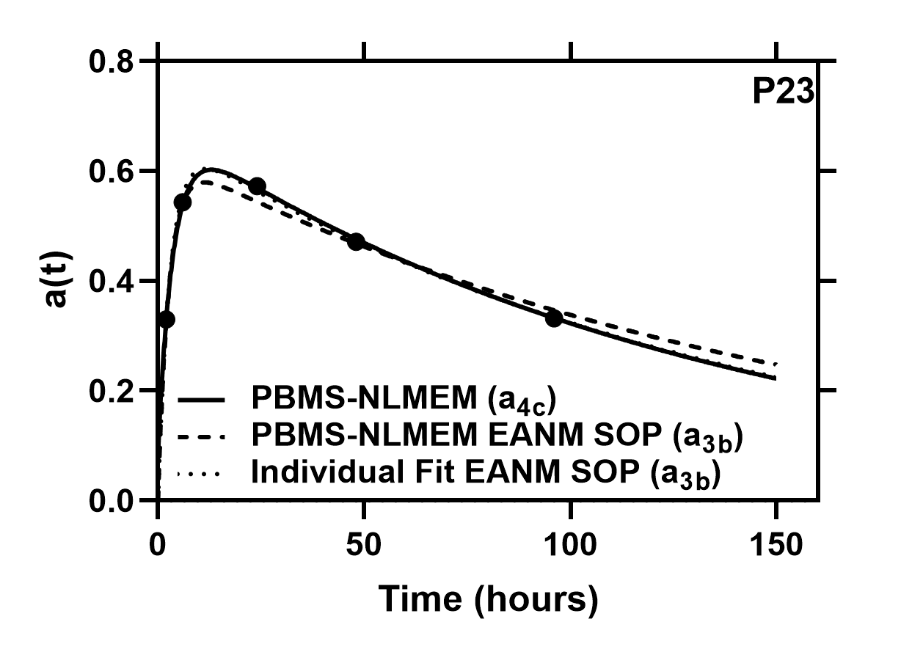

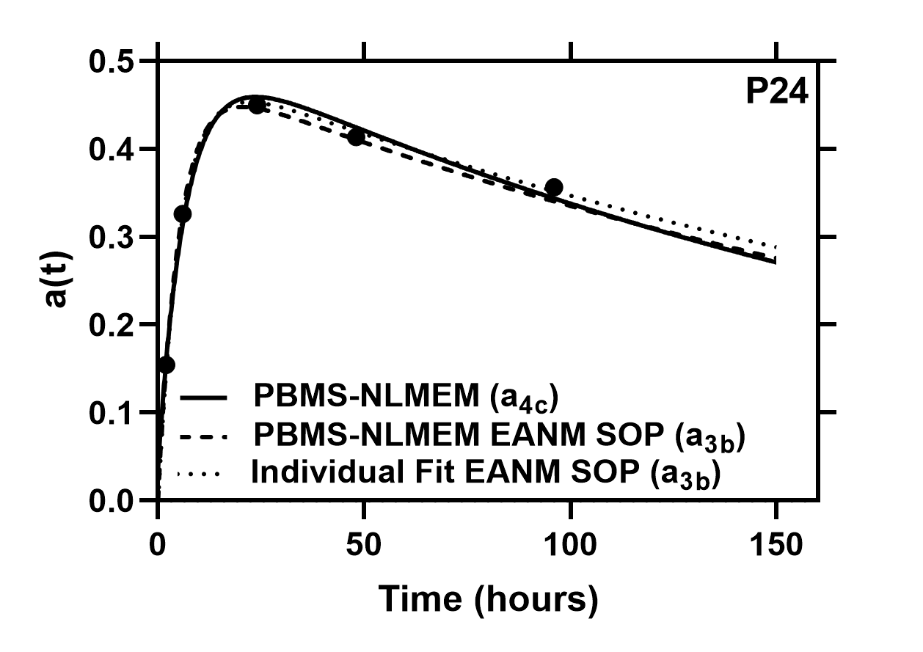

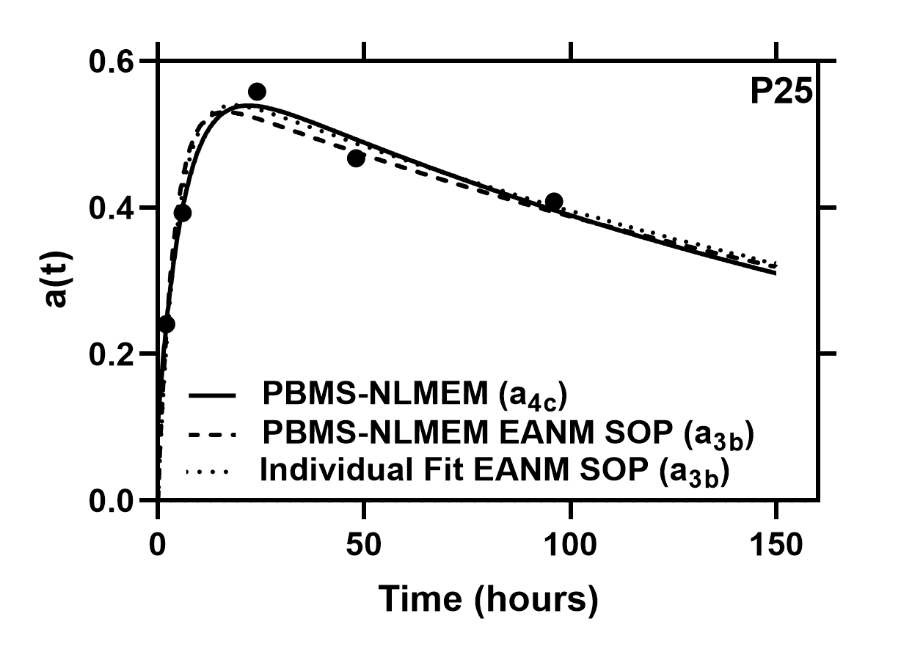

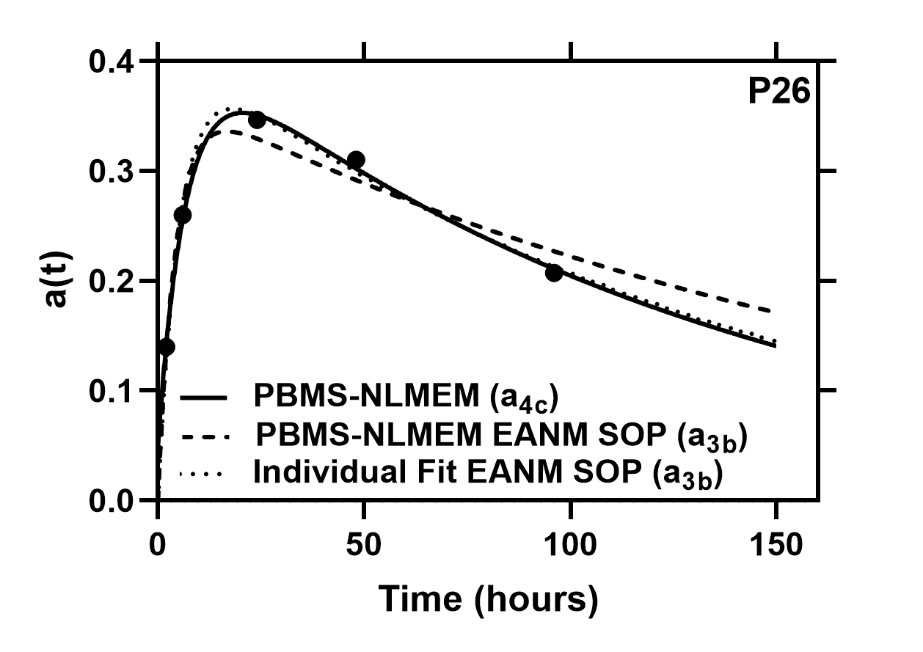

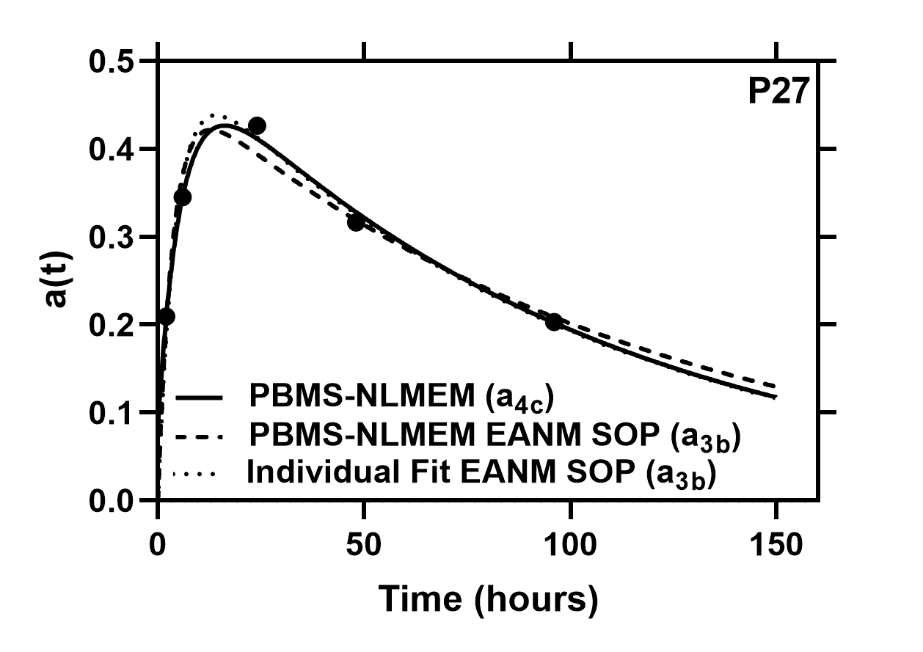

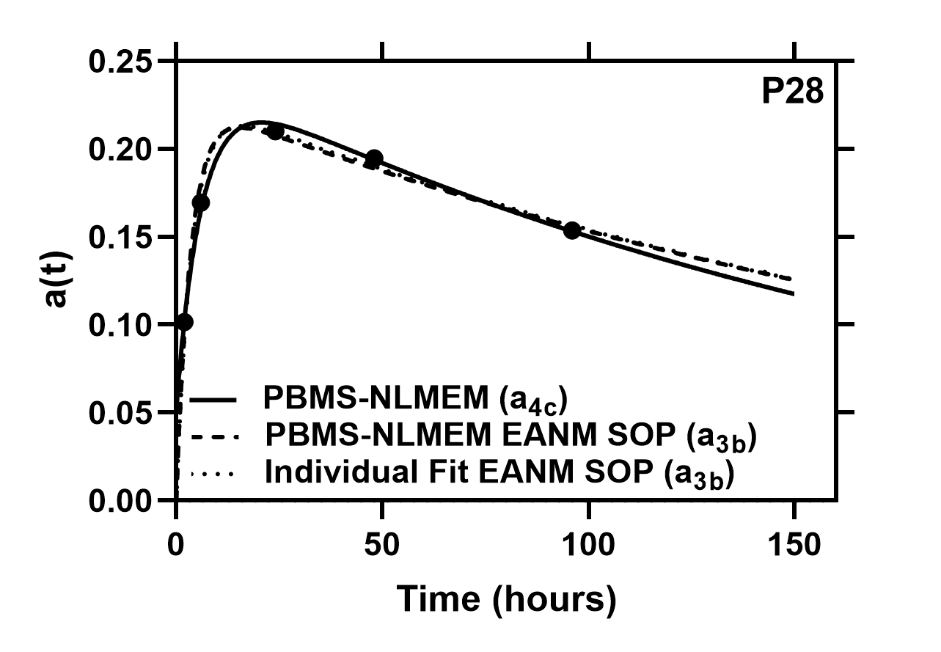

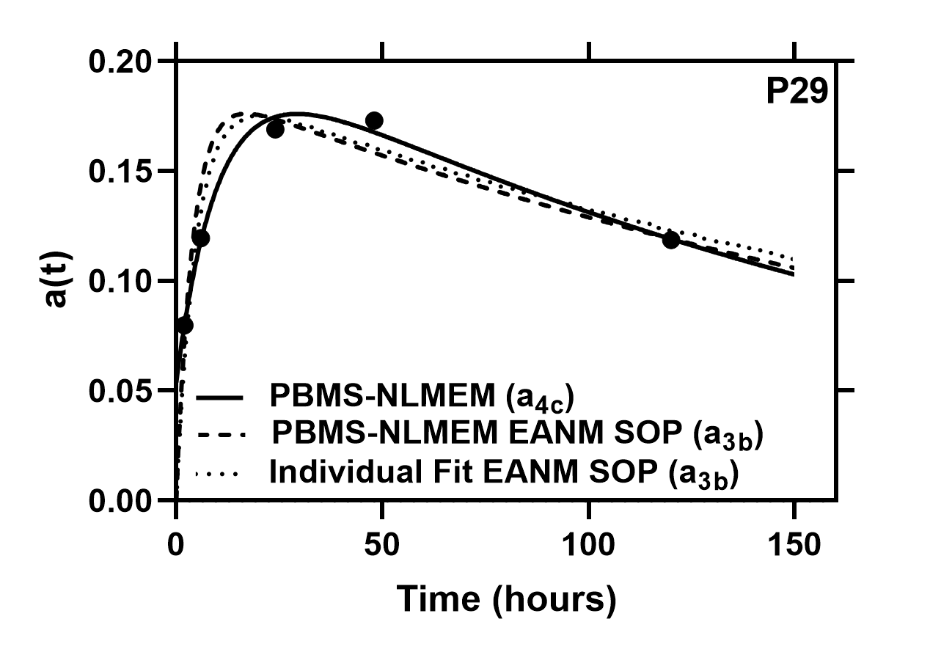

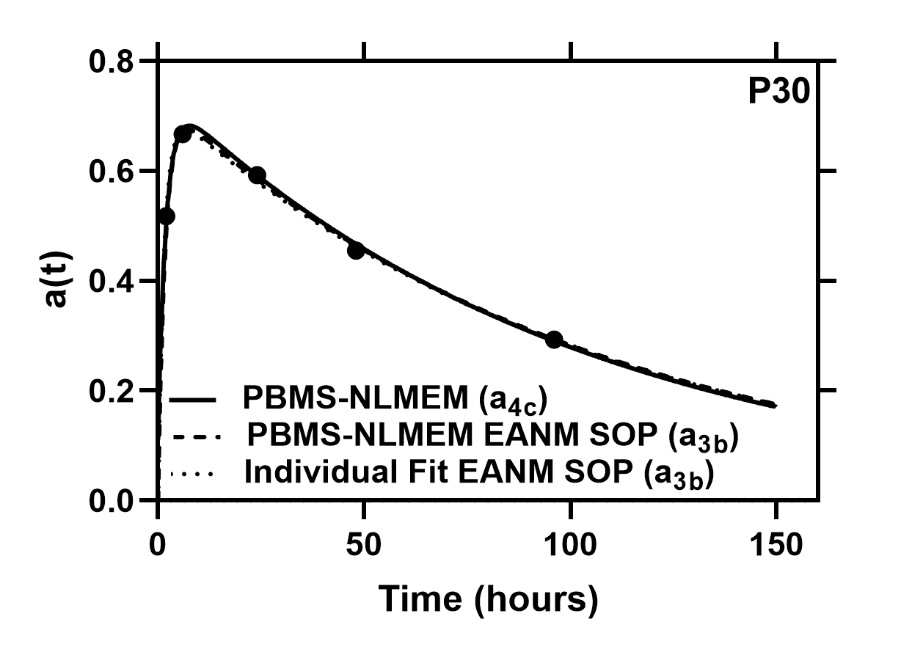

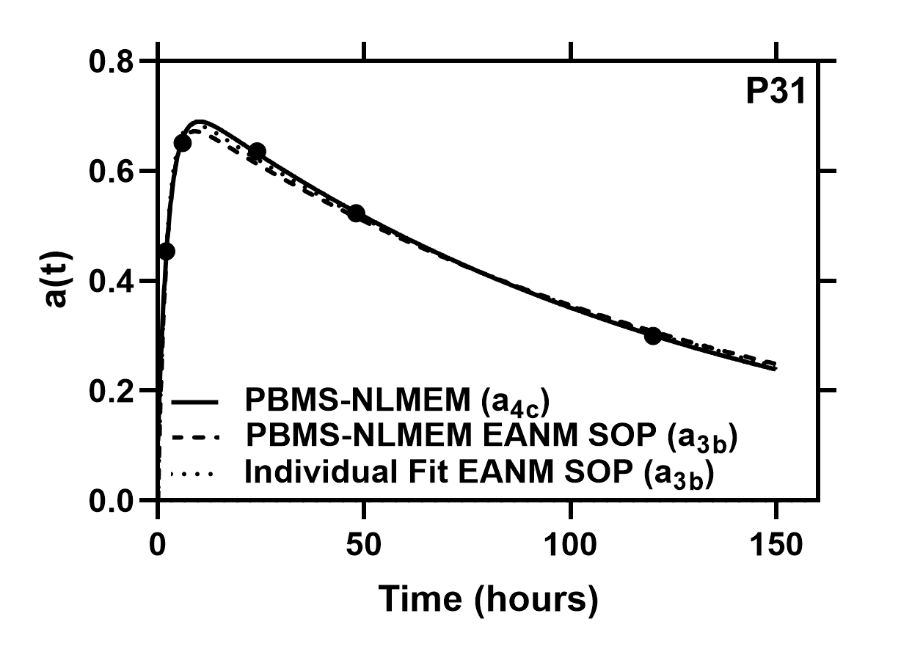

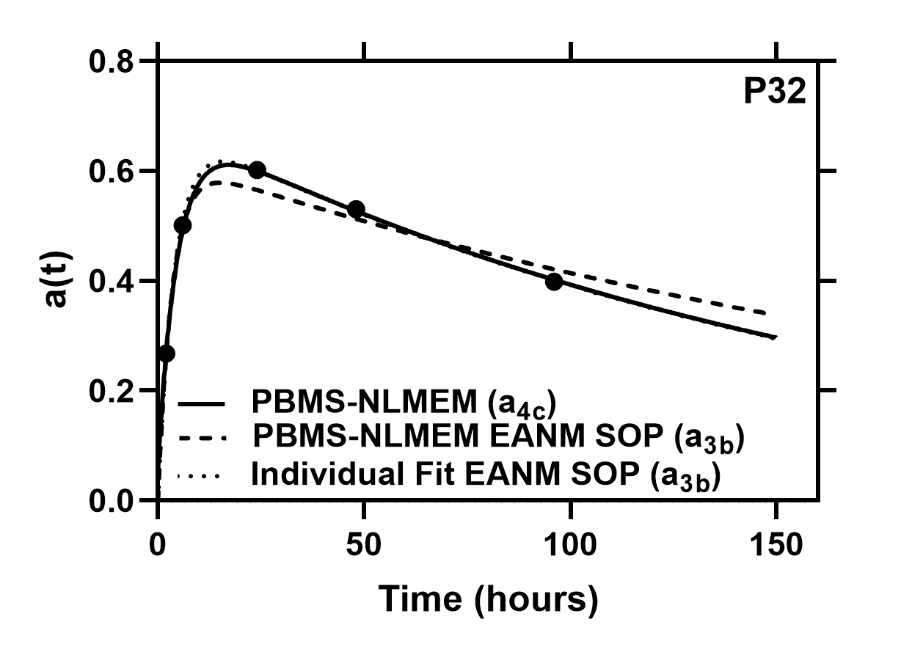

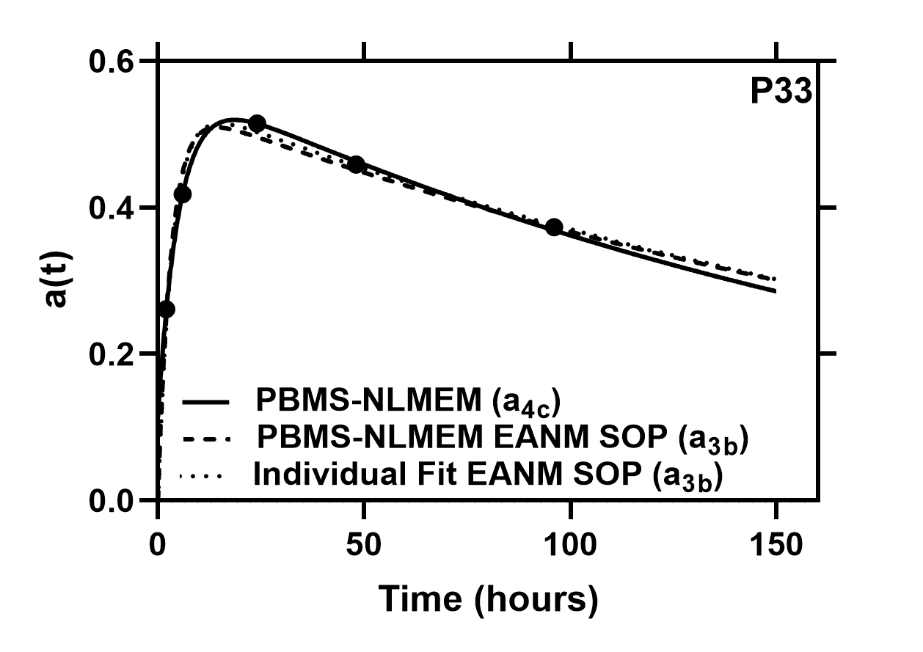

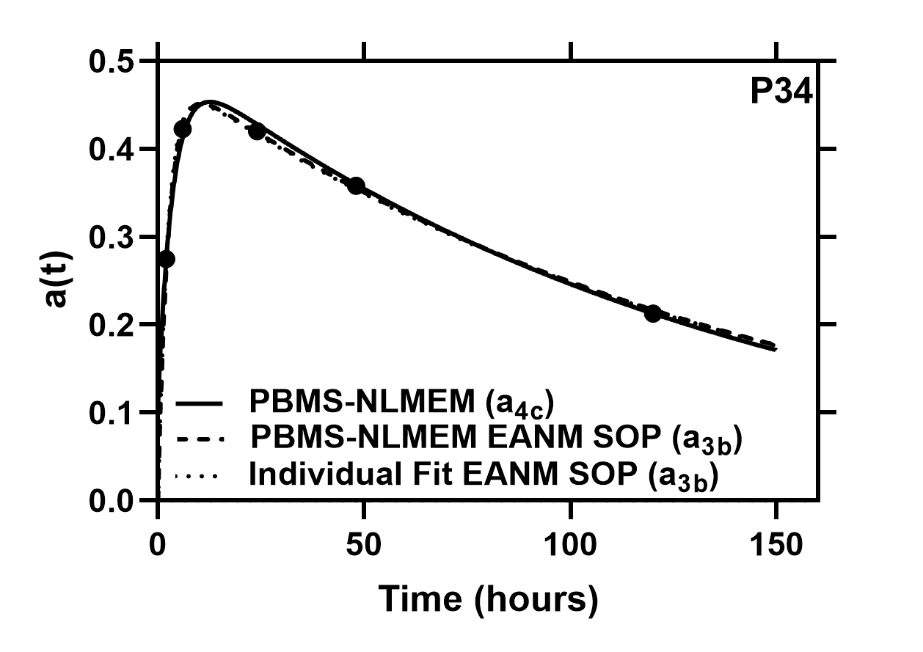

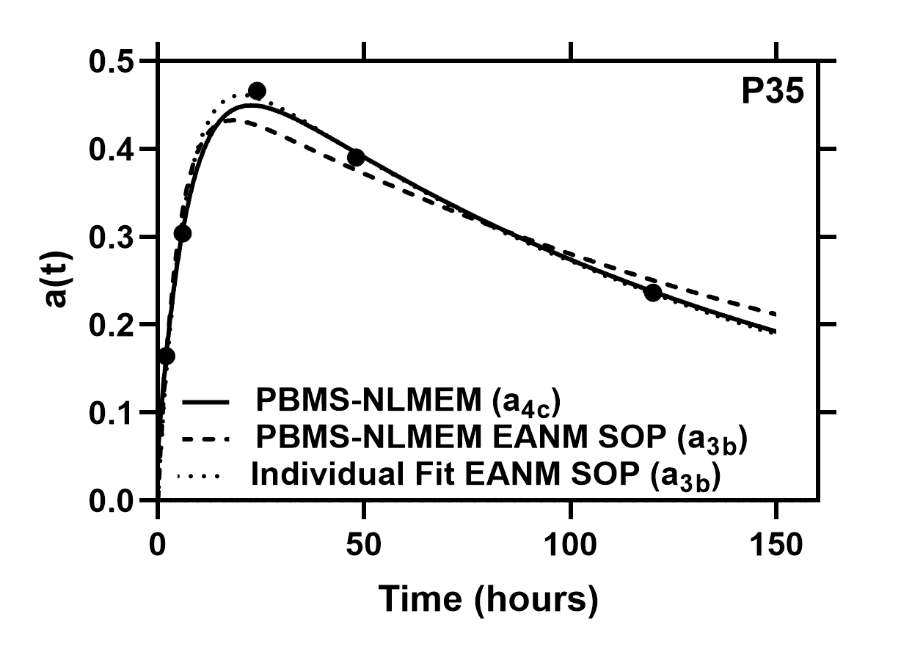

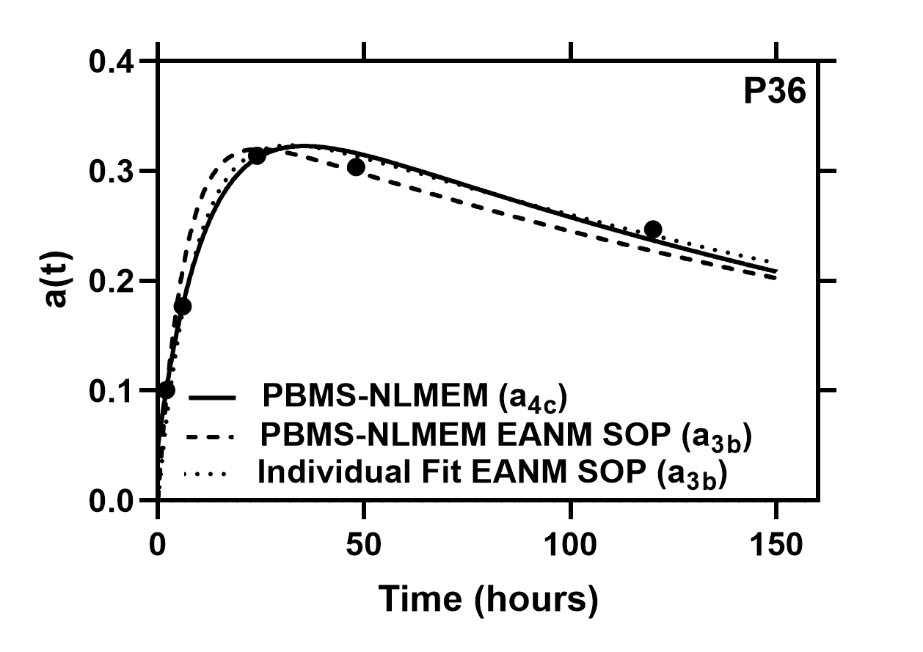

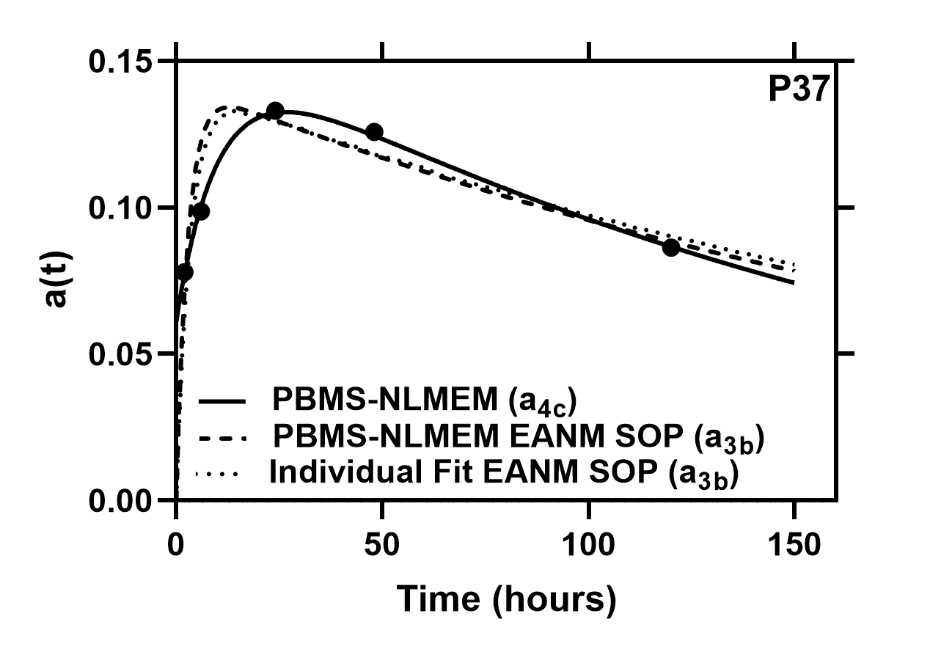

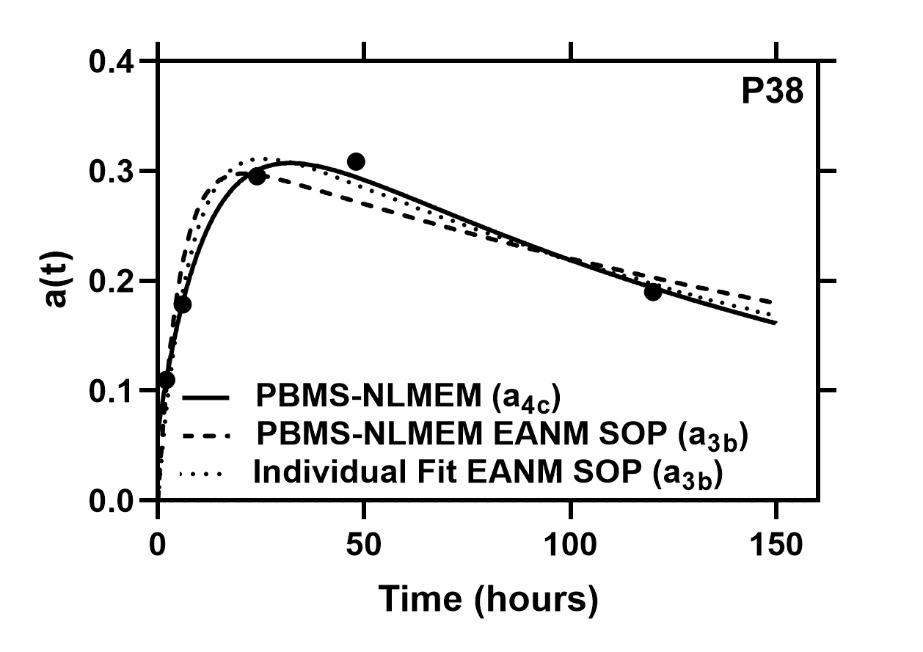

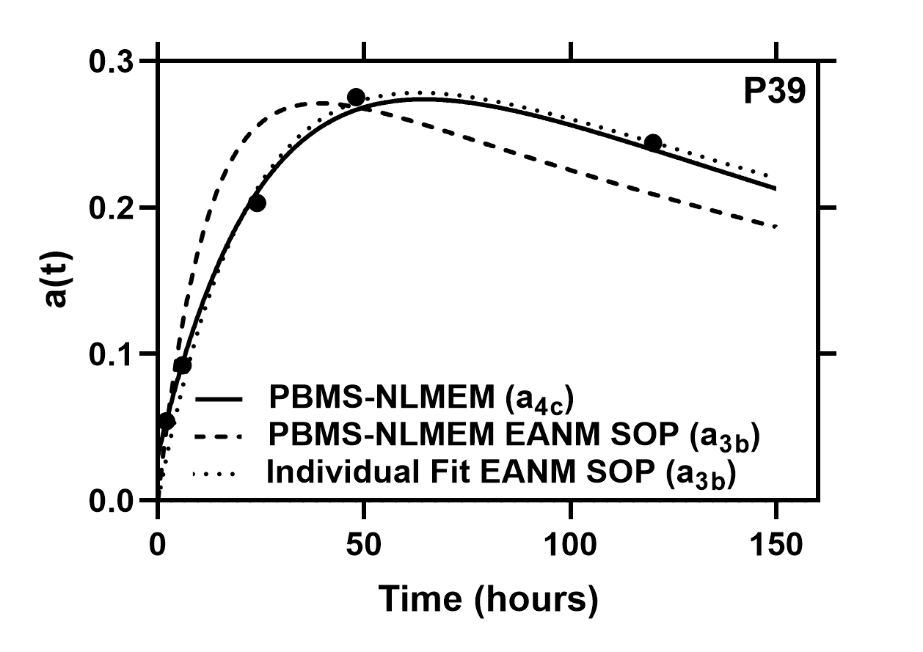

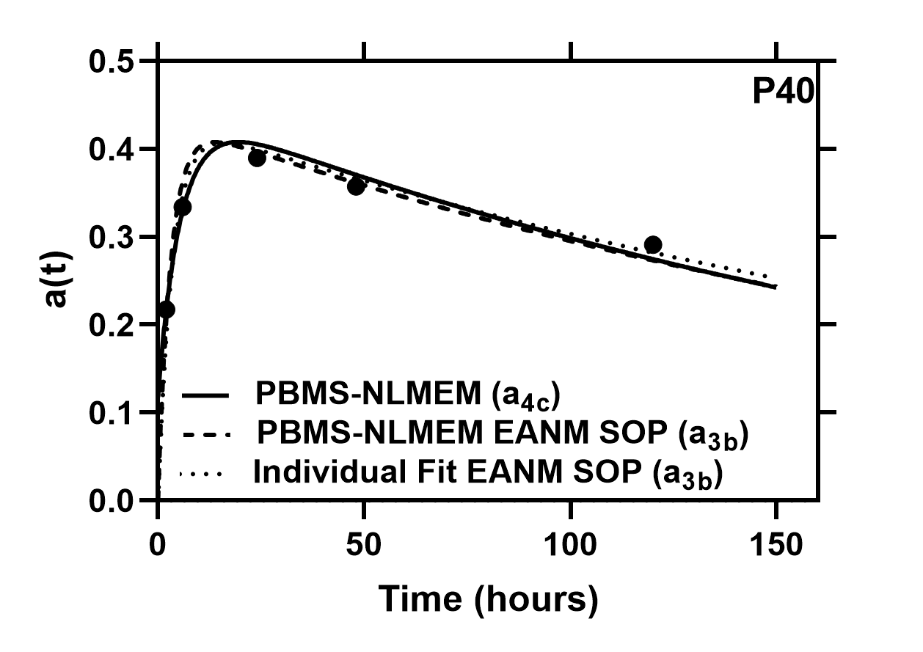

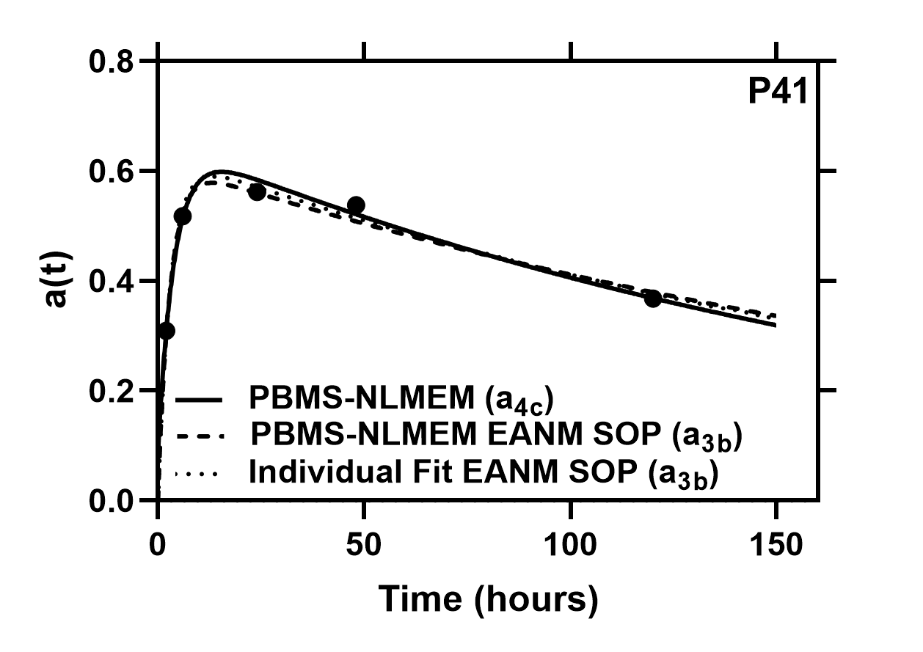

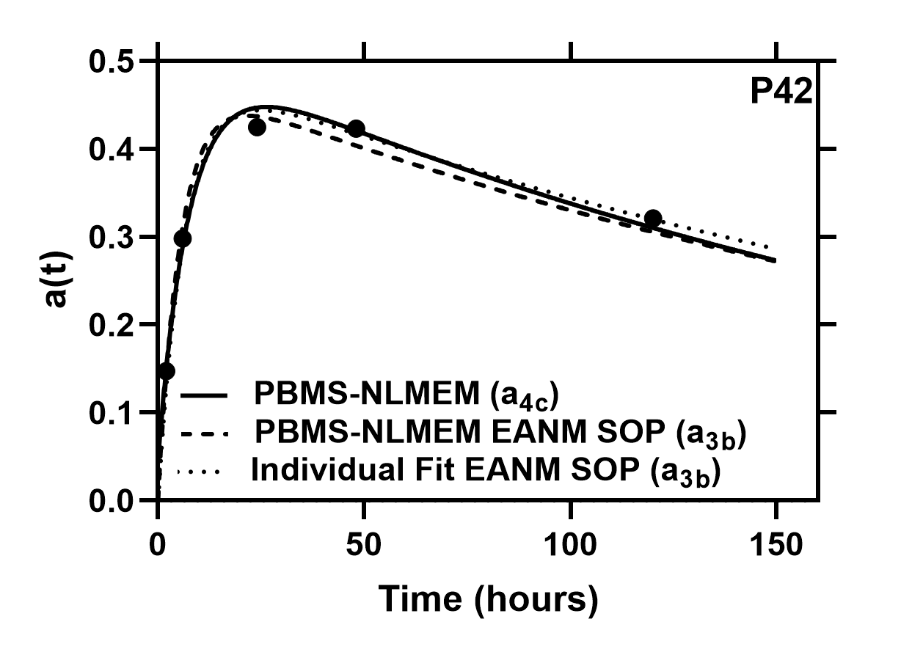

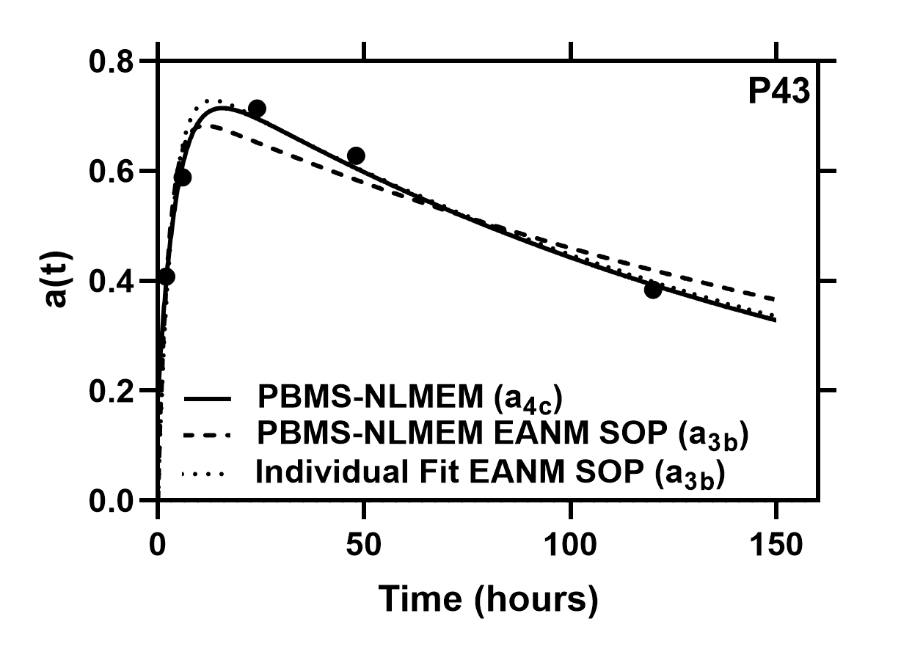

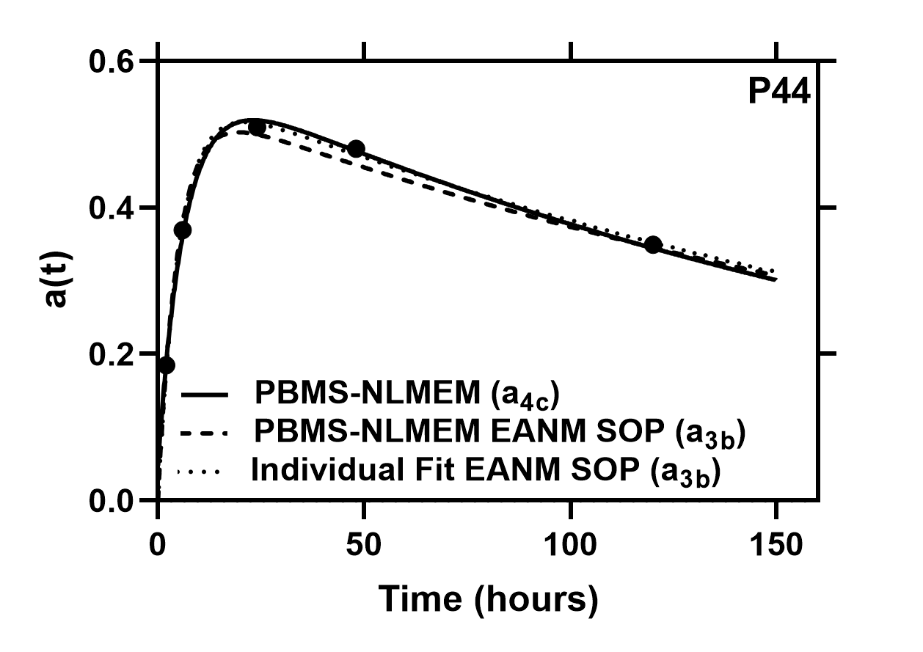

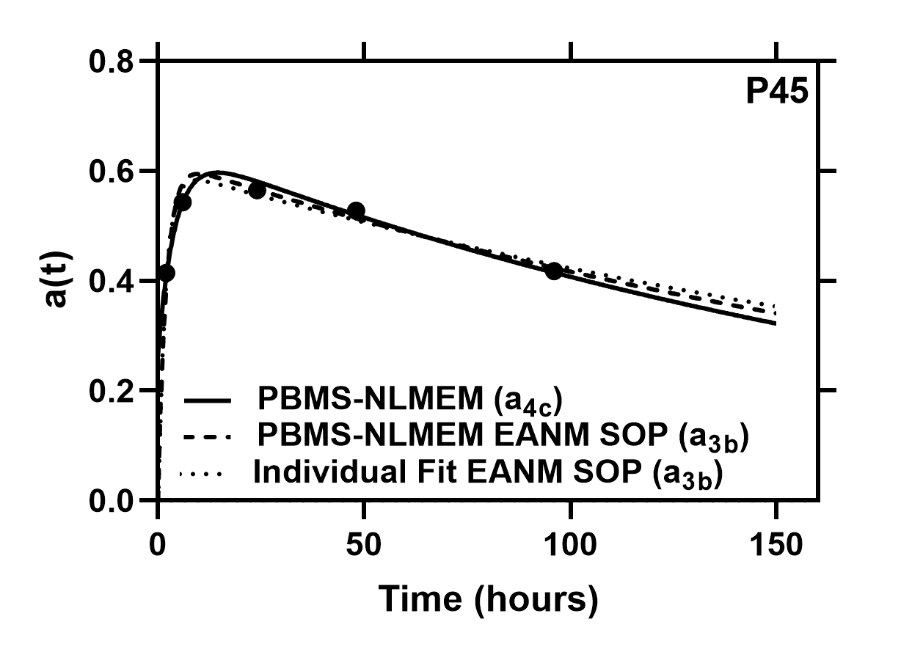

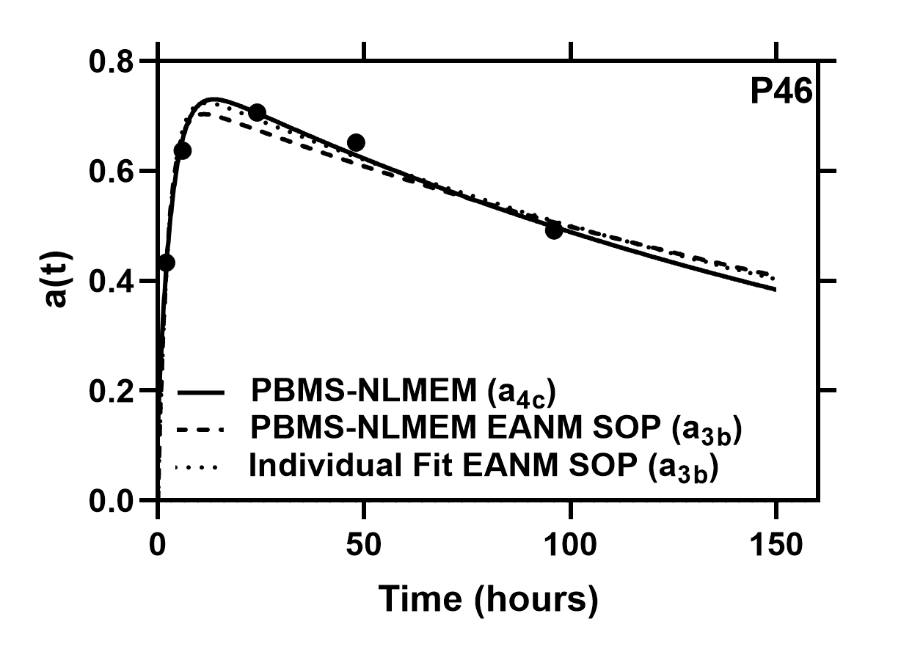

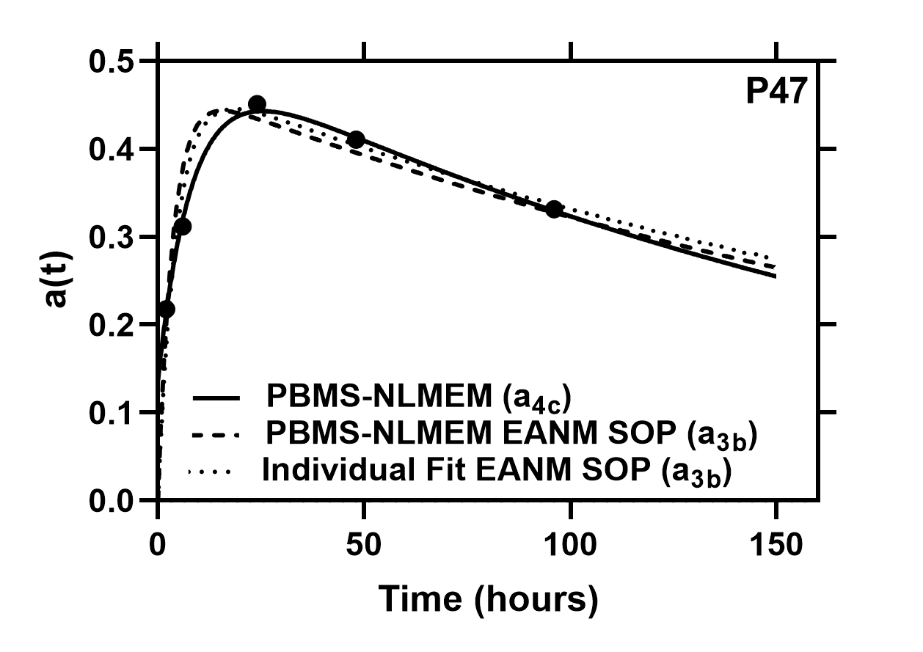

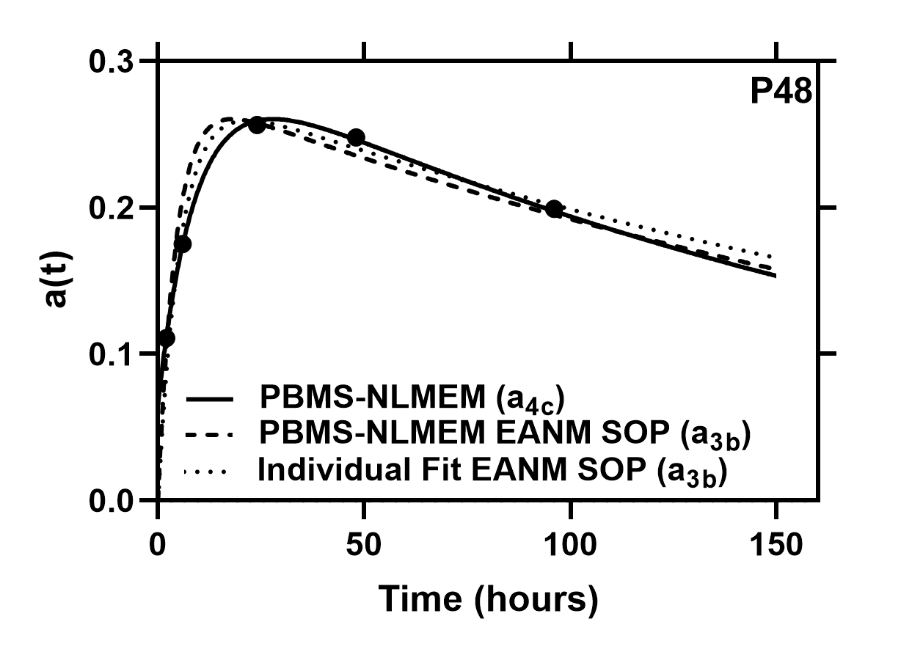

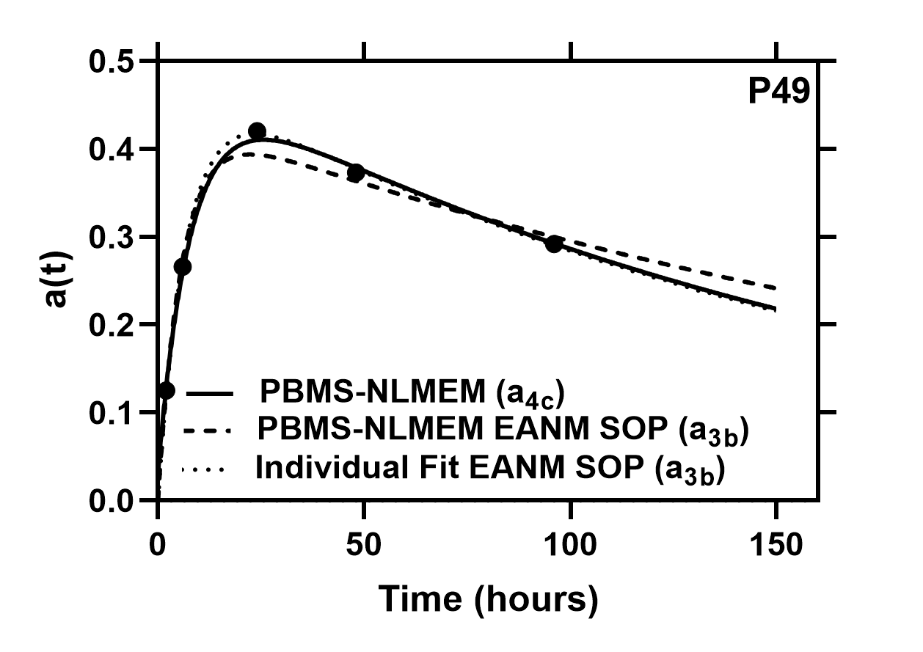

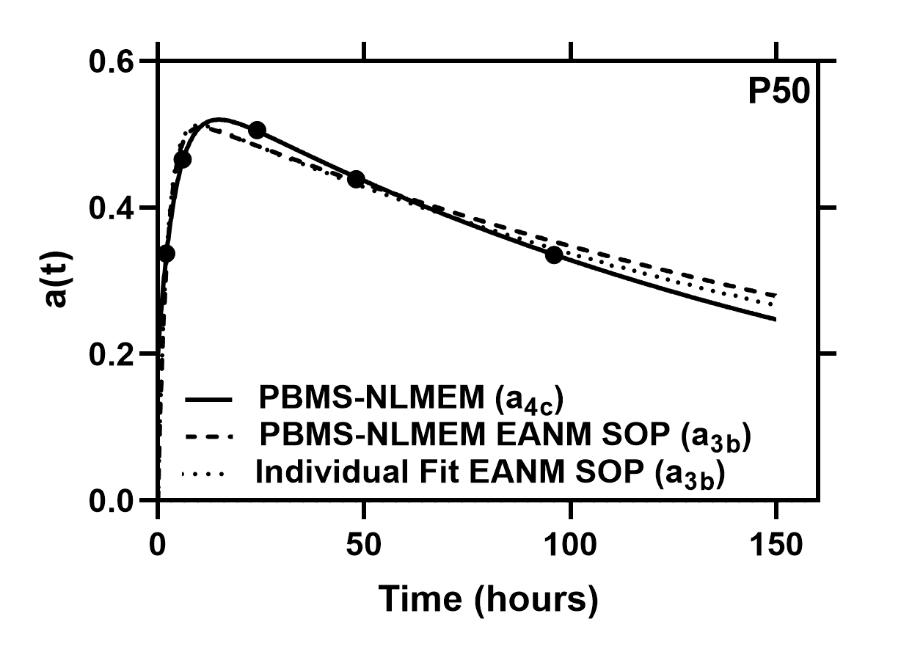

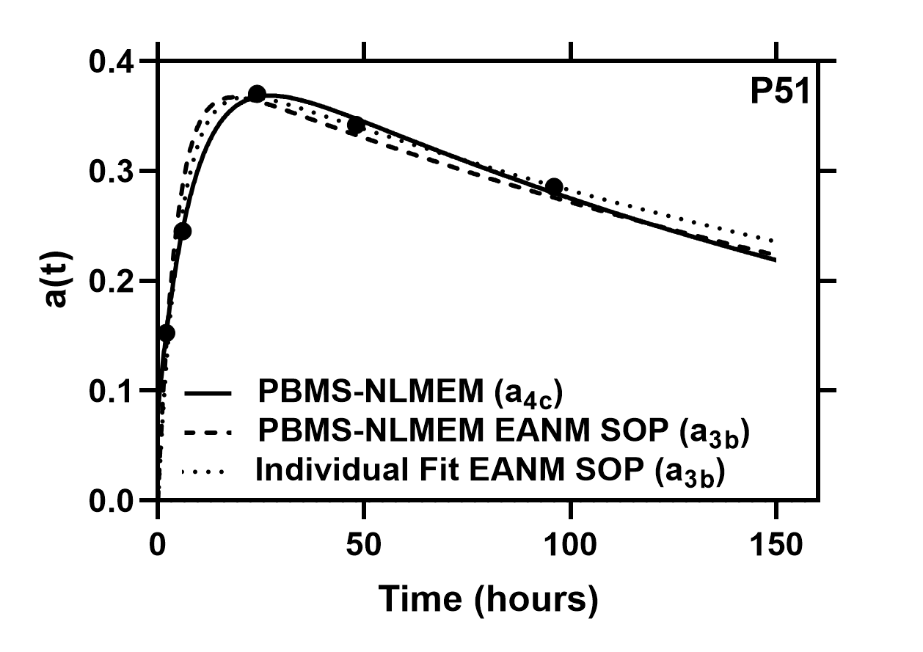

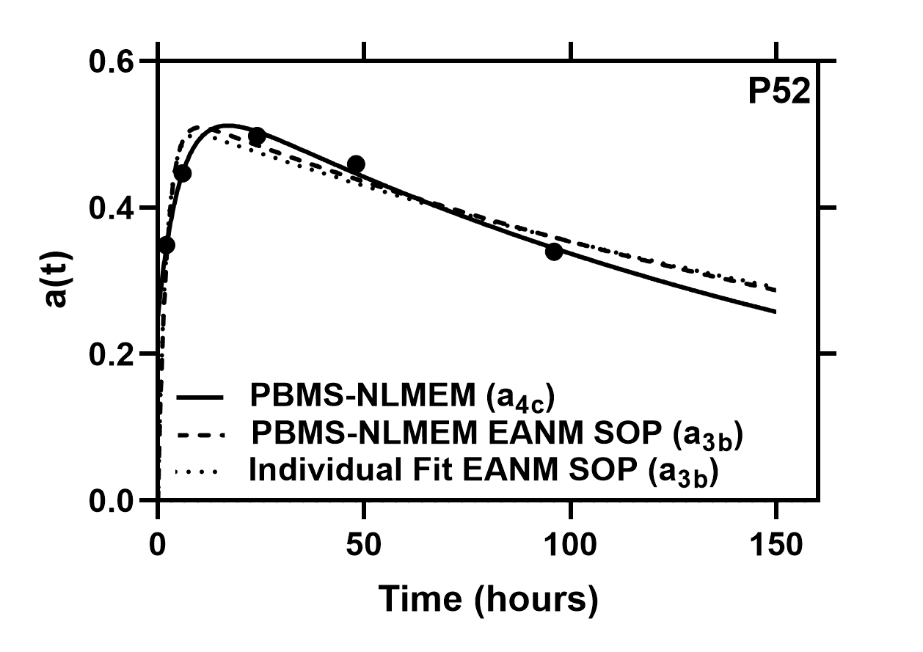

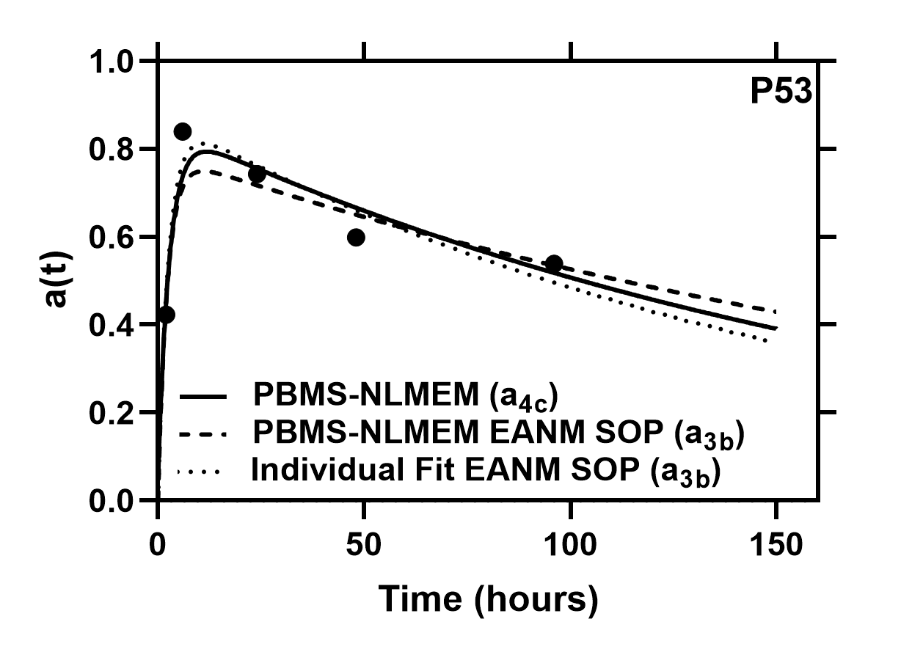

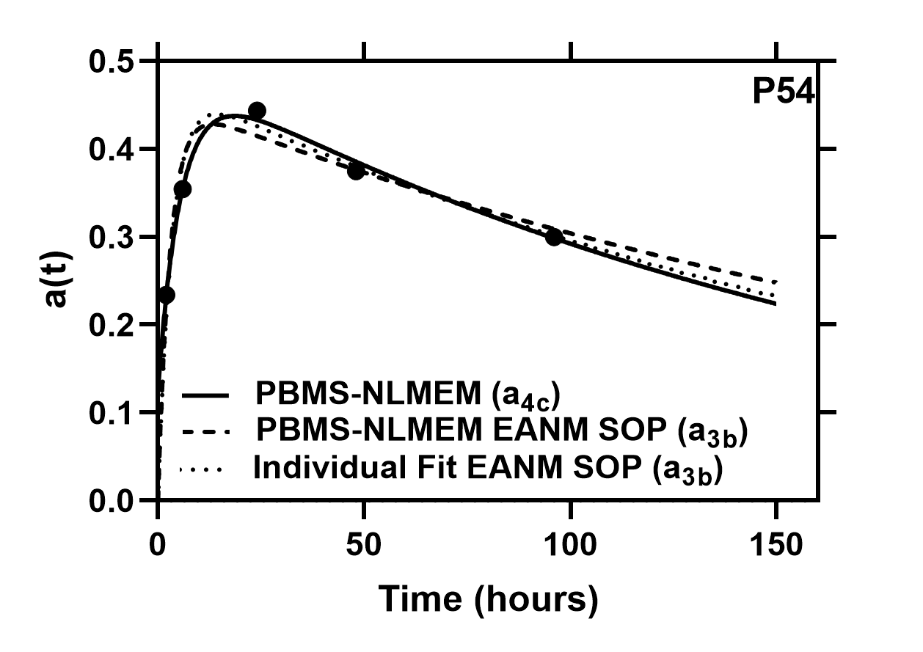

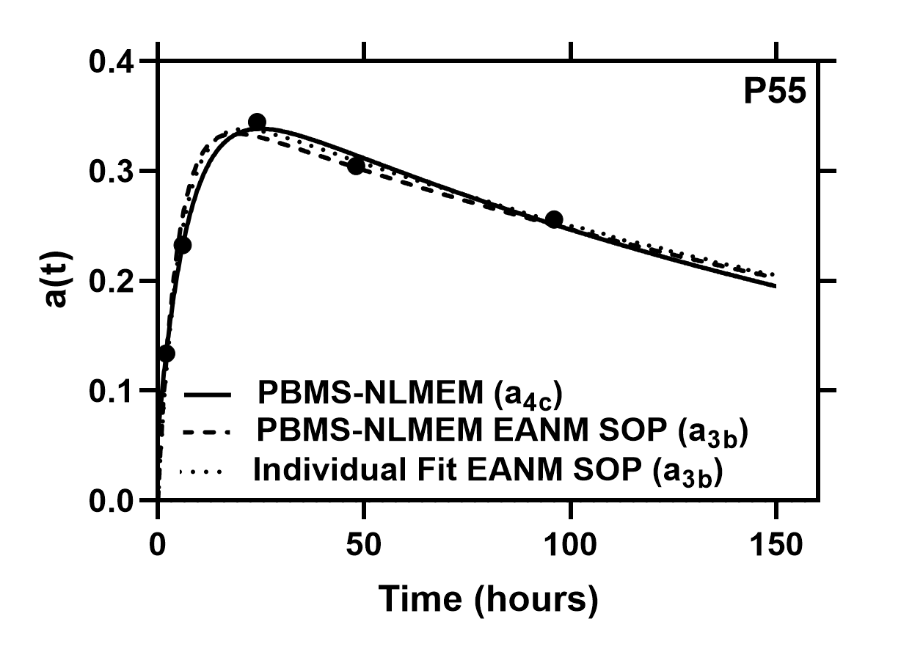

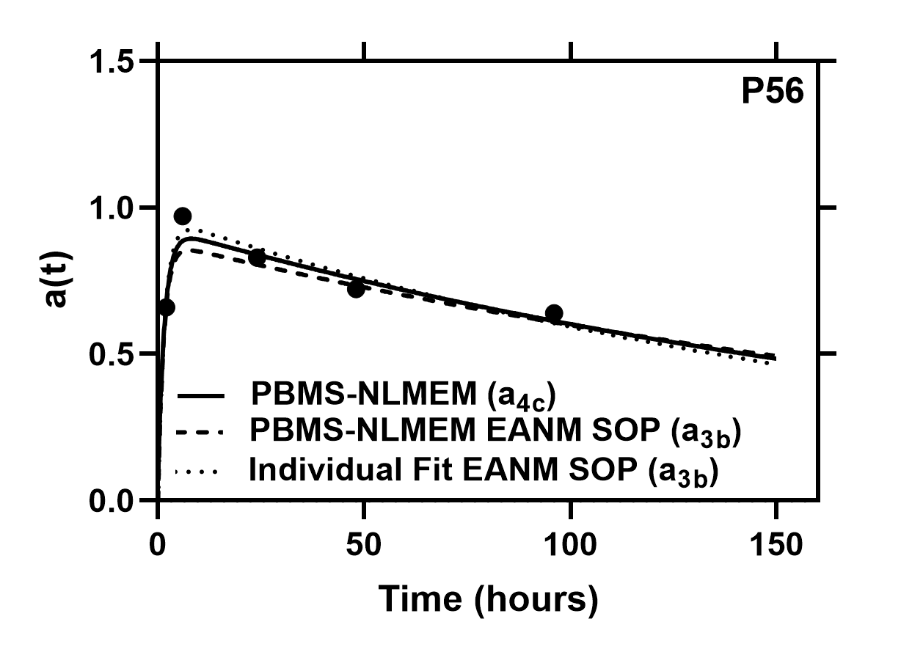

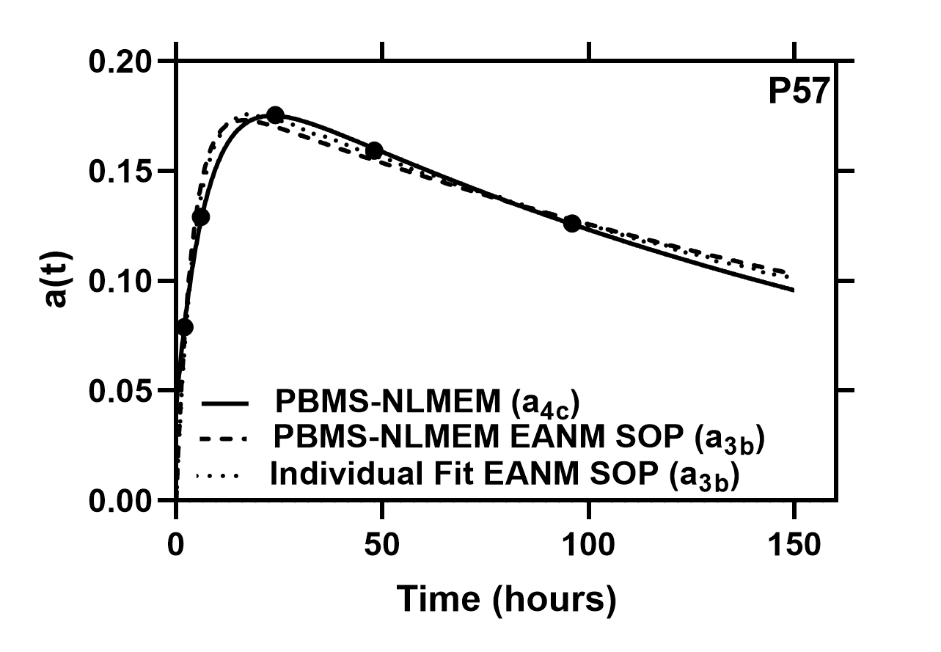

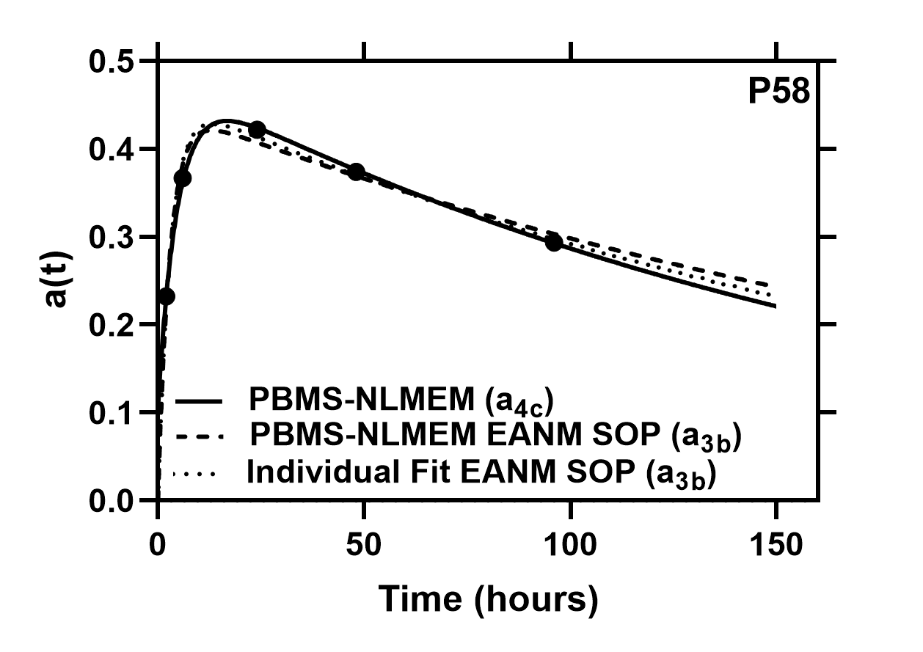

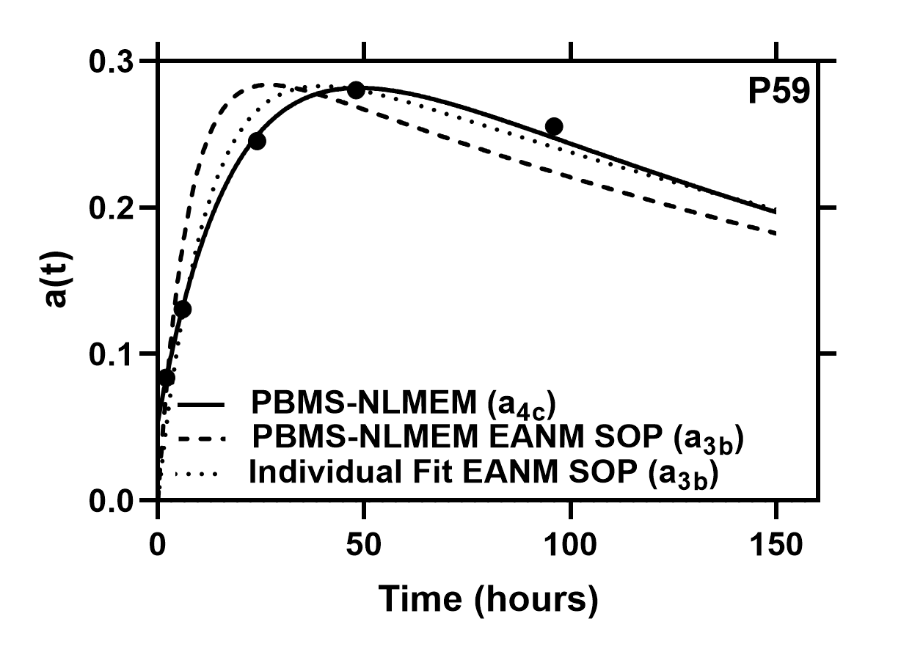

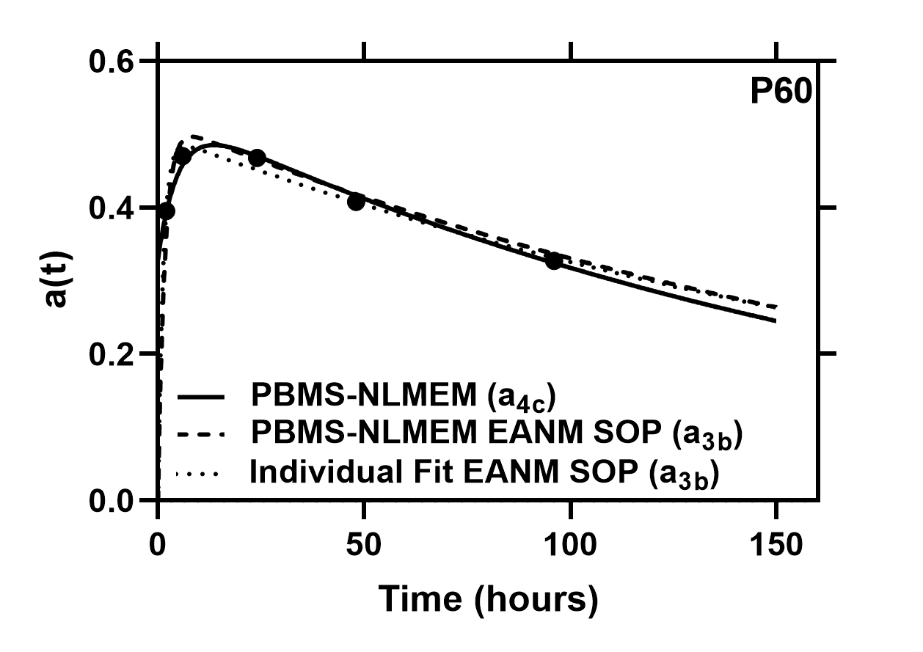

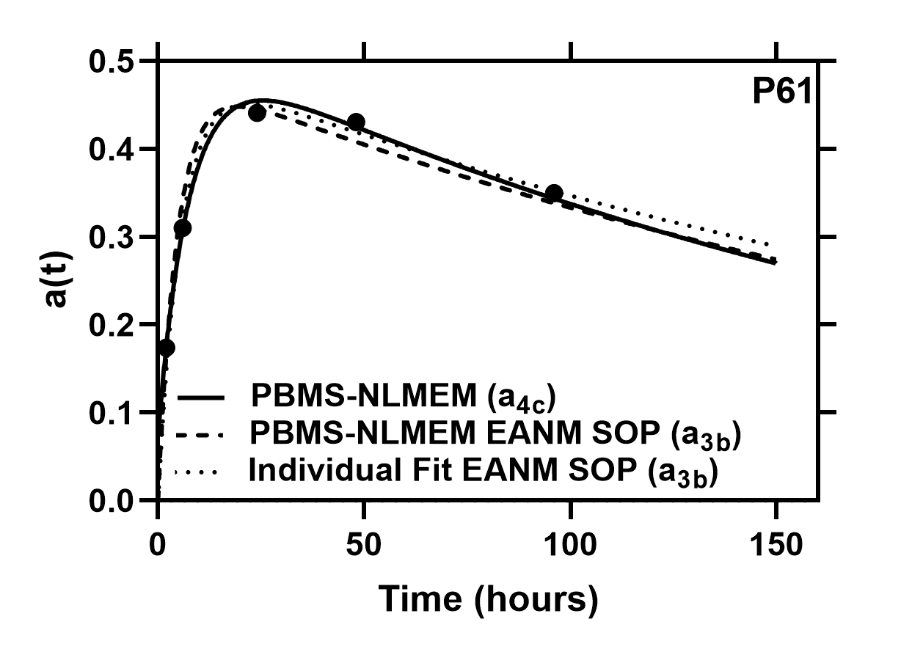

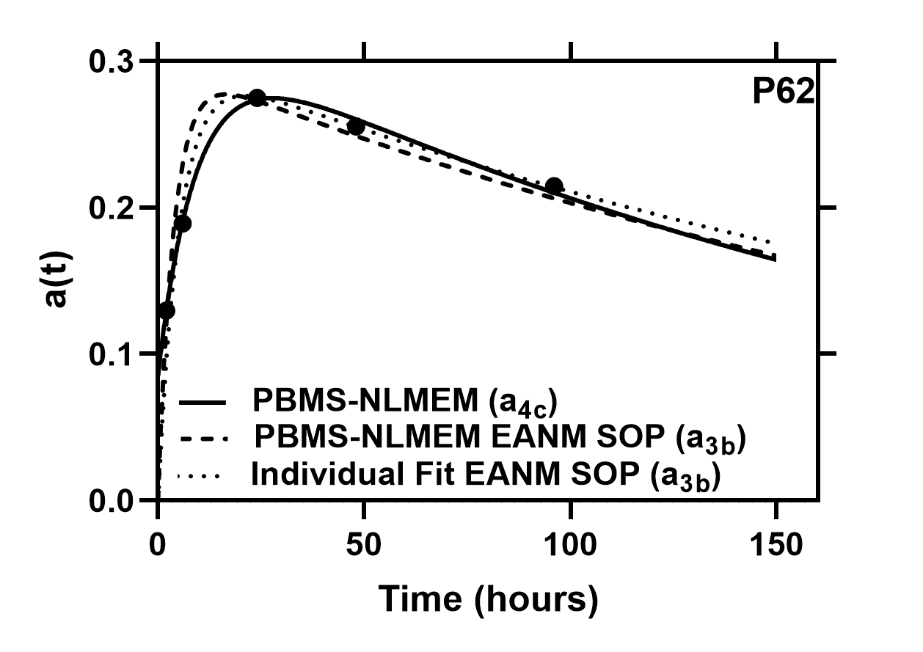

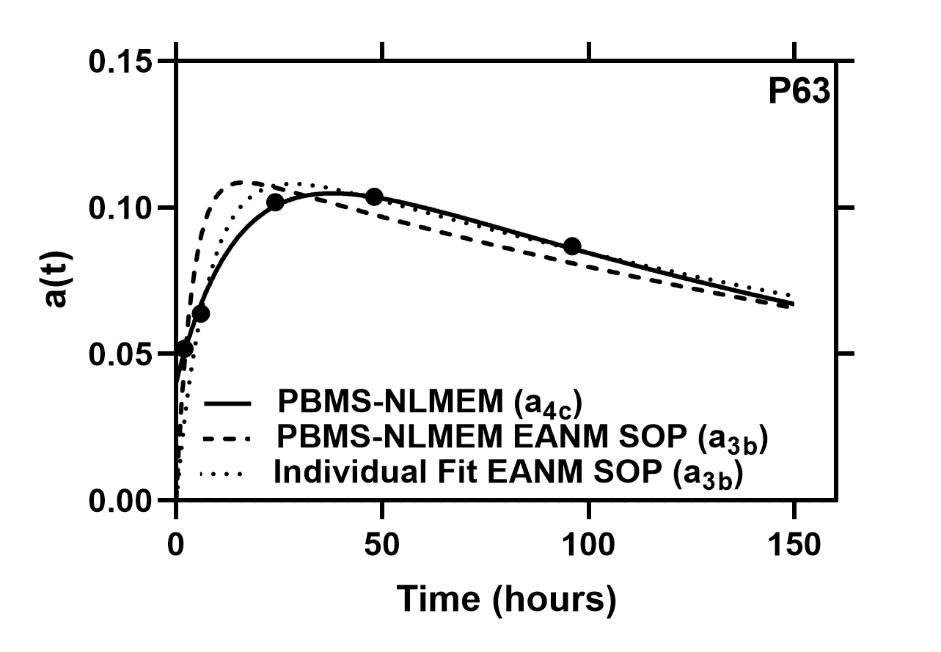

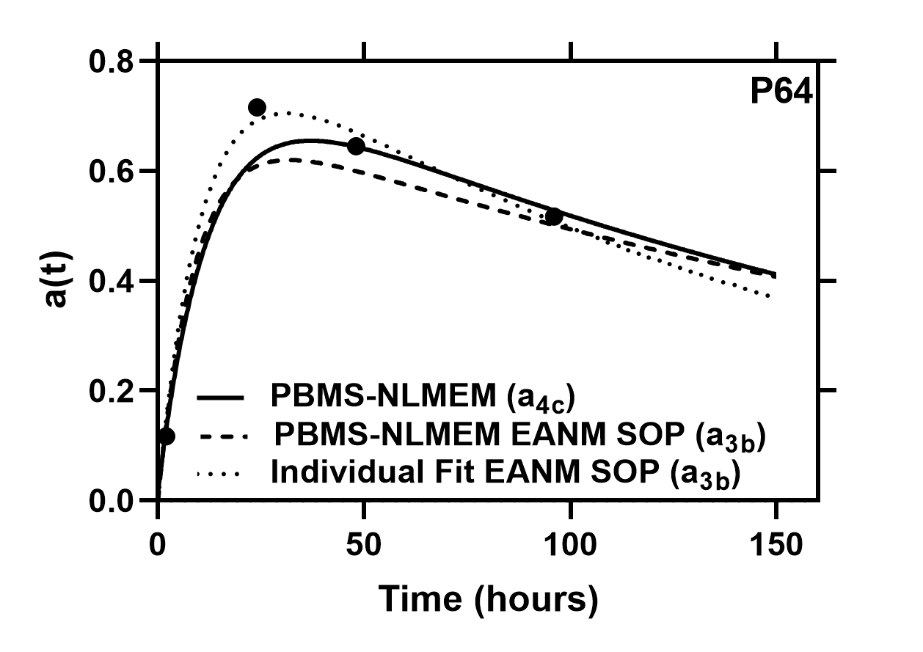

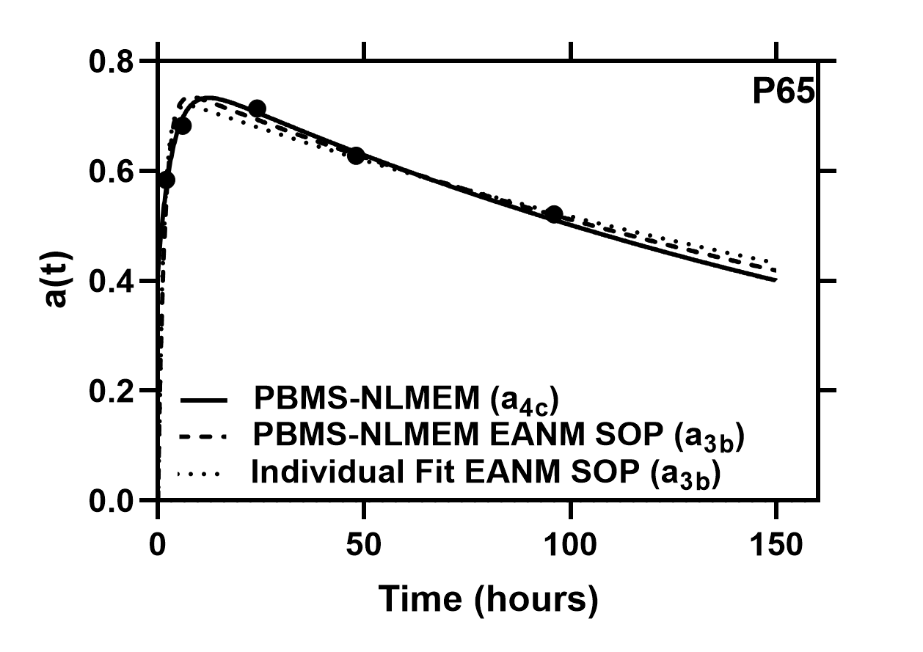

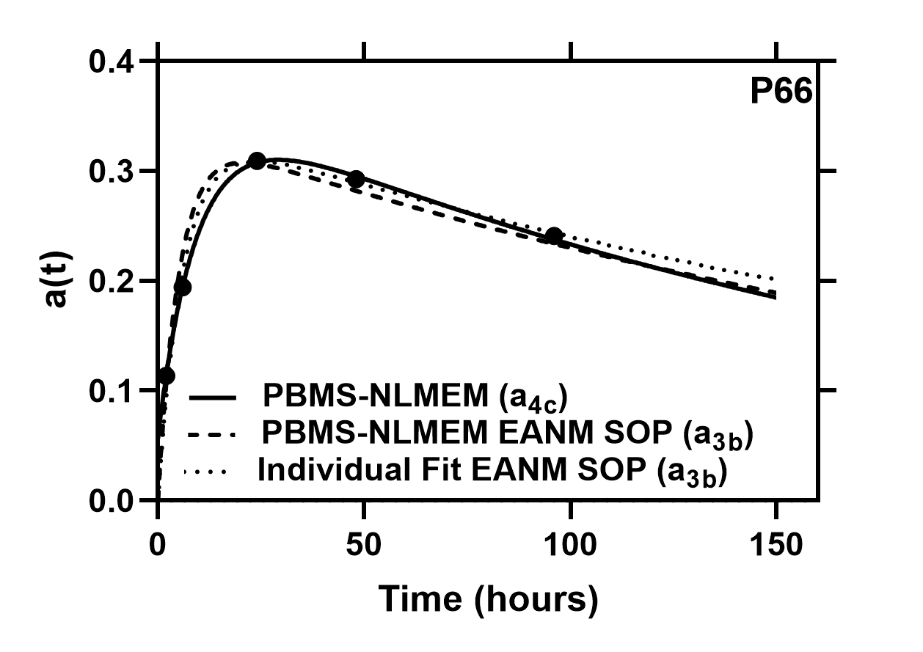

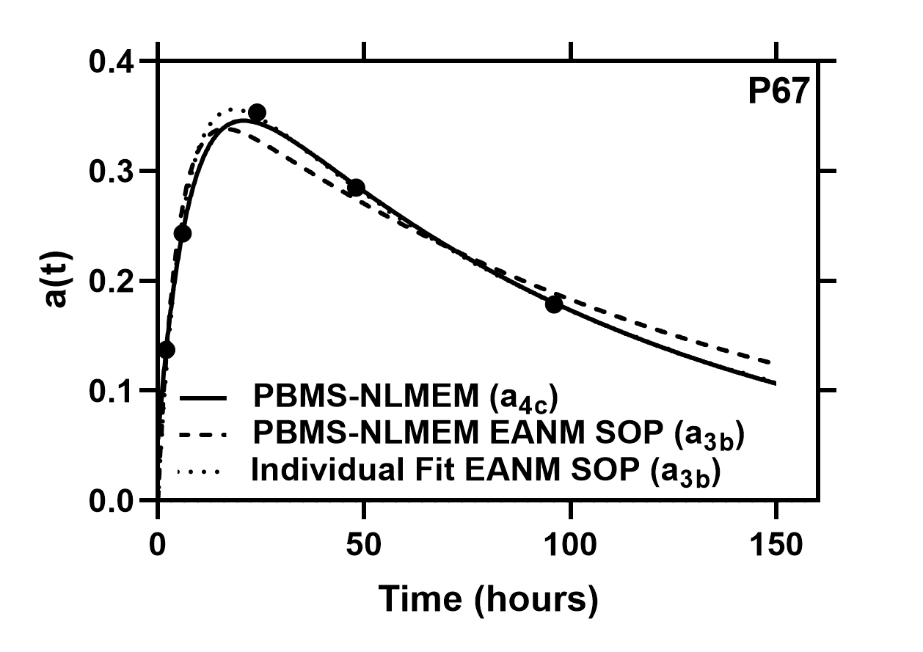

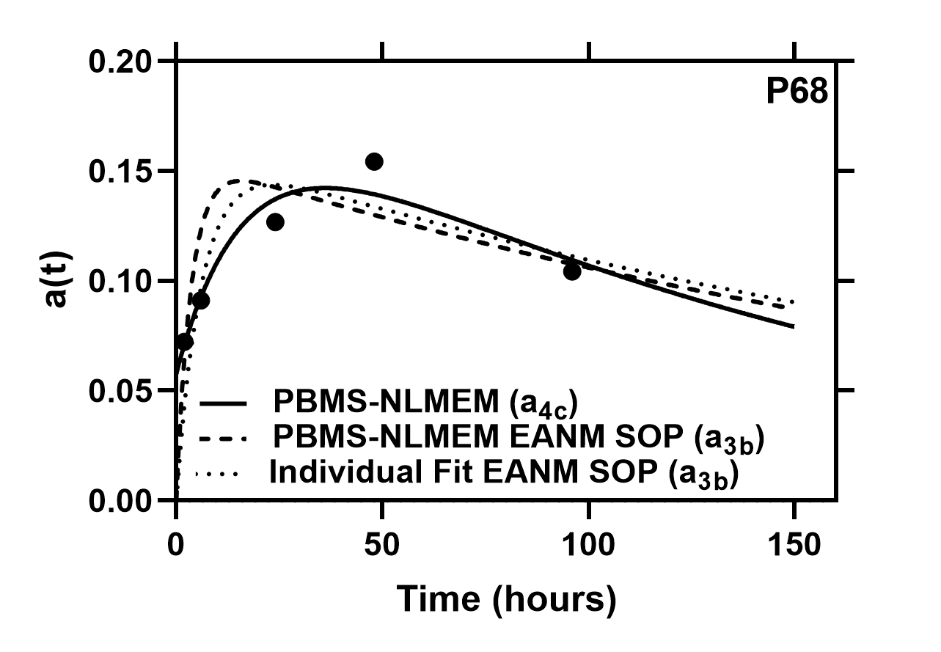

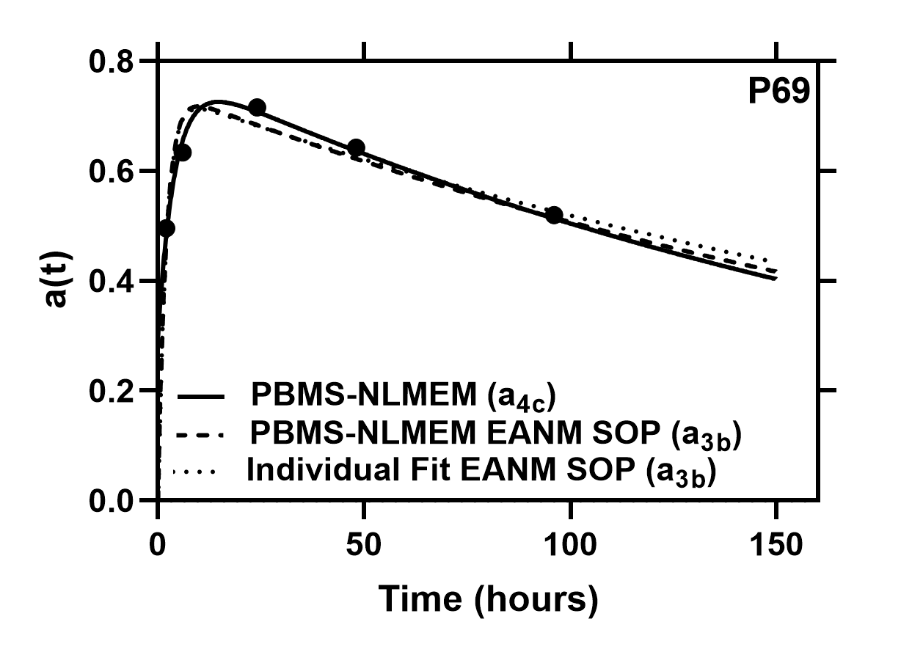

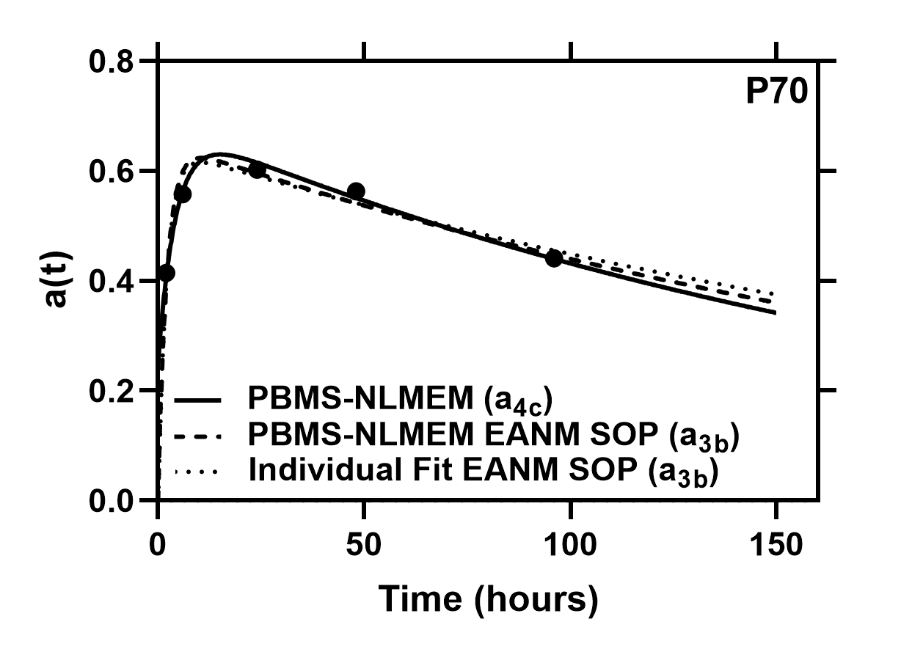

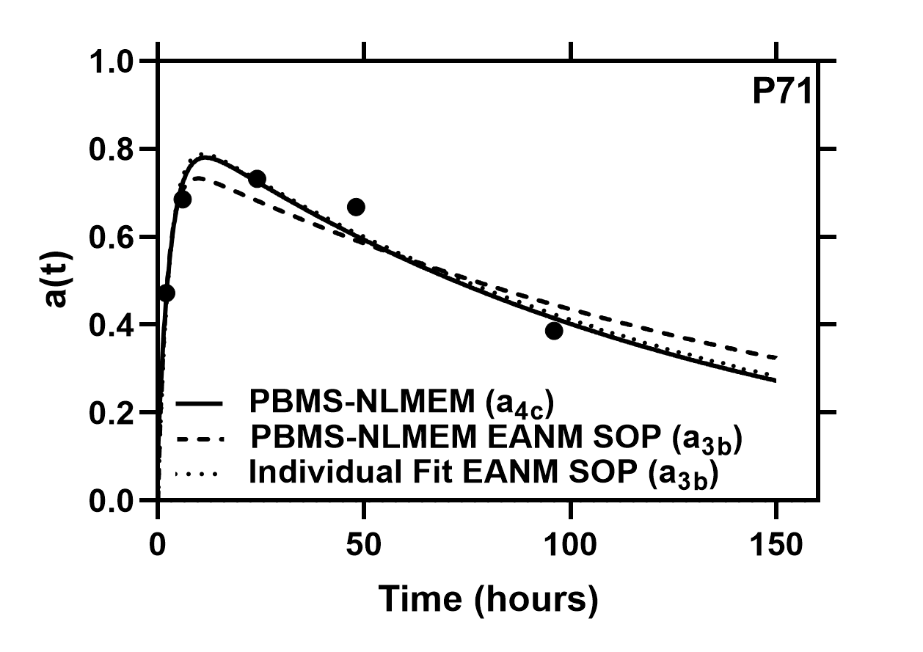

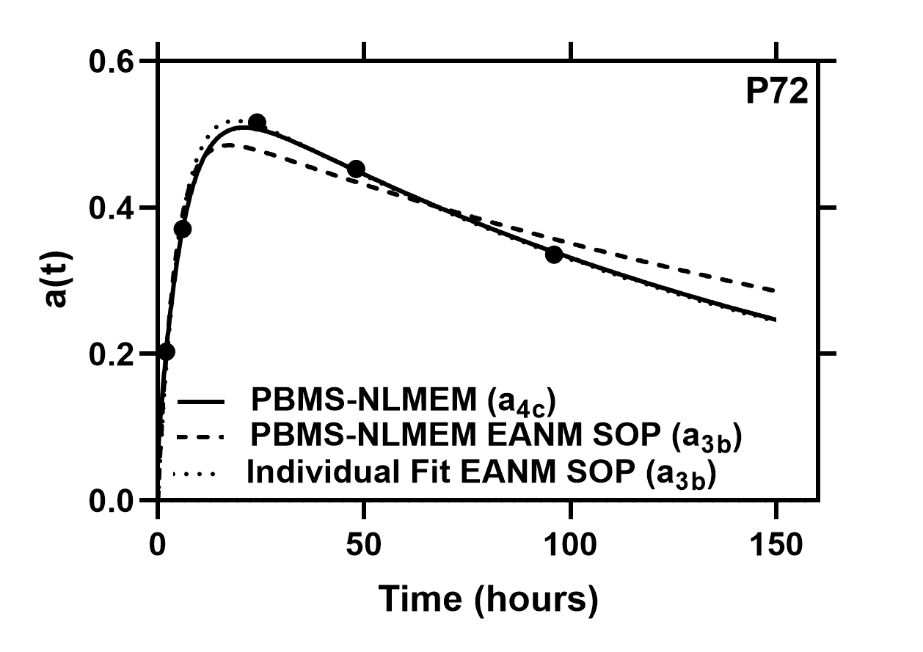

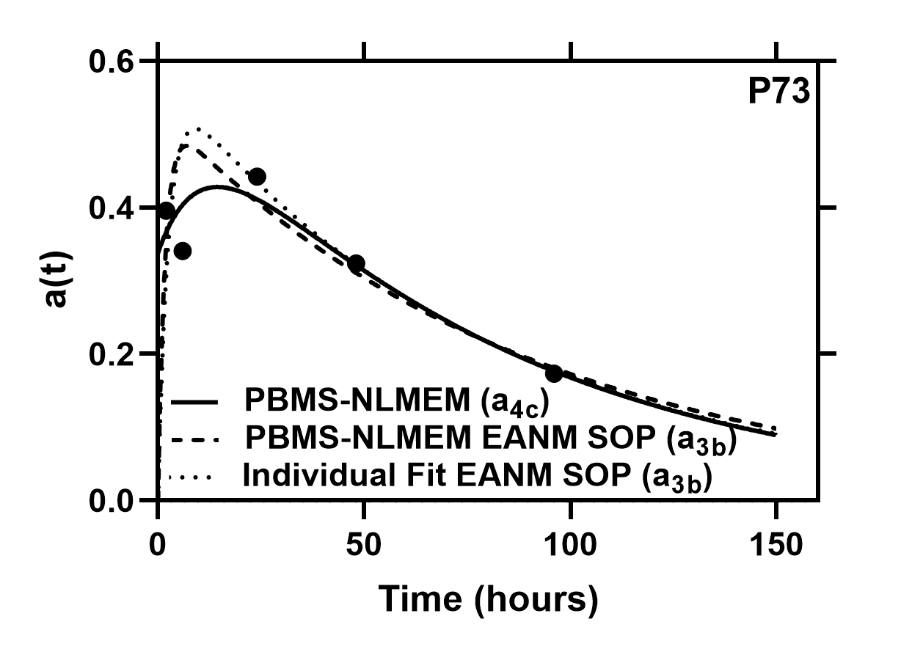


**Figure S2.** Time-activity data and fit curves using the PBMS-NLMEM ($a_{4c}$), PBMS-NLMEM EANM SOP ($a_{3b}$), and Individual Fit EANM SOP ($a_{3b}$) method

**EANM SOP Compartmental Model [2, 3]**


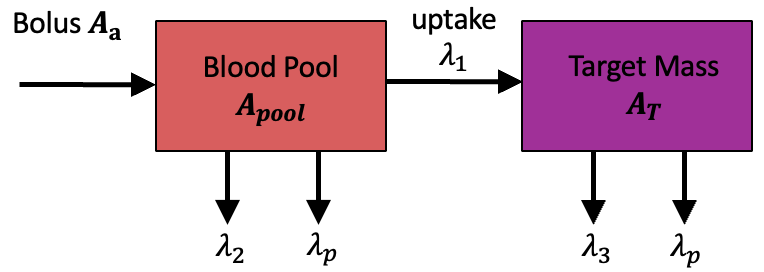


where:

$\boldsymbol{U}\left( \boldsymbol{t} \right)$: *^131^I* uptake in the Target Mass at time t

$\boldsymbol{TIA}$: Time-Integrated Activity

$\boldsymbol{\lambda}_{\boldsymbol{1}}$: uptake rate into the target mass

$\boldsymbol{\lambda}_{\boldsymbol{2}}:$ renal clearance

$\boldsymbol{\lambda}_{\boldsymbol{3}}:$ hormone excretion

$\boldsymbol{\lambda}_{\boldsymbol{p}}:$ physical decay ($\lambda_{p}$=0.0864/d for ^131^I)

Differential equations:

$\frac{ⅆA_{pool}\left( t \right)}{ⅆt}=-(\lambda_{1}+\lambda_{2}+\lambda_{p})\text{⋅}A_{pool}\left( t \right)$ (S1)

$\frac{ⅆA_{T}\left( t \right)}{ⅆt}=\lambda_{1}\text{⋅}A_{pool}\left( t \right)-$ $(\lambda_{3}+\lambda_{p}$)$\text{⋅} A_{T}\left( t \right)$ (S2)

Analytical solution

From Eq. (S1) we obtain

=>$A_{pool}\left( t \right)=A_{a}\cdotⅇ^{-(\lambda_{1}+\lambda_{2}+\lambda_{p})\cdot t}$ , (S3)

and with Eqs (S2) and (S3)

=>$A_{T}\left( t \right)=A_{a}\text{⋅}\frac{\lambda_{1}}{\lambda_{2}+\lambda_{1}-\lambda_{3}}$ $\text{⋅}\left[ ⅇ^{-\left( \lambda_{3}+\lambda_{p} \right)\text{⋅}t}-ⅇ^{-\left( \lambda_{1}+\lambda_{2}+\lambda_{p} \right)\text{⋅}t} \right]$ (S4)

Thus,

$\frac{A_{T}(t)}{A_{a}}=U\left( t \right)=\frac{\lambda_{1}}{\lambda_{2}+\lambda_{1}-\lambda_{3}}$ $\text{⋅}\left[ ⅇ^{-\left( \lambda_{3}+\lambda_{p} \right)\text{⋅}t}-ⅇ^{-\left( \lambda_{1}+\lambda_{2}+\lambda_{p} \right)\text{⋅}t} \right]$ (S5)

Calculation of the TIA for the thyroid within the EANM SOP compartmental model

$TIA=\int_{0}^{\infty} U\left( t \right)dt=$ $\frac{\lambda_{1}}{\lambda_{2}+\lambda_{1}-\lambda_{3}}$ $\text{⋅}\left[ \frac{1}{\lambda_{3}+\lambda_{p}}-\frac{1}{\lambda_{1}+\lambda_{2}+\lambda_{p}} \right]$

$TIA=$ $\frac{\lambda_{1}}{\left( \lambda_{3}+\lambda_{p} \right)\left( \lambda_{1}+\lambda_{2}+\lambda_{p} \right)}$ (S6)

Description of the best model $a_{4c}\left( t \right)$ in this study

$a_{4c}\left( t \right)=\frac{\lambda_{1}}{\lambda_{2}+\lambda_{1}-\lambda_{3}}\left( e^{-\left( \lambda_{3}+\lambda_{phys} \right)t}- e^{-\left( \lambda_{1}+\lambda_{2}+\lambda_{phys} \right)t} \right)+a_{1}e^{-\left( \lambda_{1}+\lambda_{2}+\lambda_{phys} \right)t}$ (S7)

Sum-of-exponentials function (SOEF) $a_{4c}\left( t \right)$ (S7) is the SOP EANM equation (S5) with an additional term. The extra term, i.e. $(a_{1}\cdotⅇ^{-(\lambda_{1}+\lambda_{2}+\lambda_{phys})\cdot t})$, is a contribution of the blood pool (Equation (S3)) with factor $a_{1}$ which takes into account any residual count rate from the tissue activity (blood pool activity). Thus, although the measurement over the thigh was subtracted before to correct for the blood pool activity in the thyroid measurement, the correction was too small as the fitted value of $a_{1}$ is larger than zero.

References

1. Owen, J.S. and J. Fiedler-Kelly, *Introduction to population pharmacokinetic/pharmacodynamic analysis with nonlinear mixed effects models*. 2014, Hoboken, New Jersey: Wiley.

2. Hänscheid, H., et al., *EANM Dosimetry Committee series on standard operational procedures for pre-therapeutic dosimetry II. Dosimetry prior to radioiodine therapy of benign thyroid diseases.* Eur J Nucl Med Mol Imaging, 2013. **40**(7): p. 1126-34.

3. Hänscheid, H., M. Lassmann, and C. Reiners, *Dosimetry prior to I-131-therapy of benign thyroid disease.* Z Med Phys, 2011. **21**(4): p. 250-7.
